# Pesticide Residues, Gut Microbiota, and Gastrointestinal Outcomes: A Scoping Review of Human Evidence

**DOI:** 10.1111/1541-4337.70622

**Published:** 2026-08-23

**Authors:** Chengqian Ye, Qingjie Wu, Zhaochu Wang, Cai Zhang

**Affiliations:** ^1^ Department of Gastroenterology The Affiliated People's Hospital of Fujian University of Traditional Chinese Medicine Fuzhou China; ^2^ Research Base of Traditional Chinese Medicine Syndrome Fujian University of Traditional Chinese Medicine Fuzhou China; ^3^ Department of Anorectal Surgery The Affiliated People's Hospital of Fujian University of Traditional Chinese Medicine Fuzhou China; ^4^ Department of Health and Kinesiology University of Illinois Urbana‐Champaign Urbana Illinois USA; ^5^ Beckman Institute for Advanced Science and Technology University of Illinois Urbana‐Champaign Urbana Illinois USA

**Keywords:** dietary exposure assessment, glyphosate, gut microbiota, inflammatory bowel disease, neonicotinoid, organochlorine, pesticide residues, PRISMA‐ScR, scoping review, vegetables

## Abstract

Vegetables anchor healthy‐diet recommendations yet are the principal nonoccupational route by which residues of organophosphate (OP), pyrethroid, neonicotinoid, organochlorine (OC), and glyphosate herbicide enter the diet, with the gut microbiota a plausible mediator linking these residues to functional gastrointestinal (GI) and inflammatory bowel outcomes. We conducted a scoping review following the PRISMA extension for Scoping Reviews (PRISMA‐ScR), searching PubMed (2010 to April 2026) with mandatory backward and forward citation tracing for human studies of dietary or biomarker‐confirmed pesticide exposure against gut microbiota, intestinal barrier and inflammation, functional GI disorders, or inflammatory bowel disease (IBD). Risk of bias (RoB) was appraised with ROBINS‐E and Cochrane RoB 2.0, and evidence was synthesized in a 60‐cell pesticide‐class × outcome gap matrix. Twenty‐eight papers were retained; 20 full‐text‐extracted human studies entered the synthesis, populating 20 of 60 cells (33%). The strongest convergent within‐scope signal was OC exposure × IBD, where three cohorts agreed in direction (hazard ratios 1.56–1.61; Faroese risk ratio 3.04 on 37 cases). As a microbiota‐mediation exemplar for an adjacent non‐GI endpoint, the MCMC Nanjing pregnancy cohort linked a pesticide environmental risk score to gestational diabetes (adjusted odds ratio 4.5; 95% CI, 1.4–13.8; internal‐validation area under the curve 0.83), with a *Dorea*‐branch microbiota mediating part of the path. Glyphosate × IBD and neonicotinoid × any GI outcome remained empty. RoB centered on Moderate (11 of 22 rated cohorts), with six elevated‐bias contributors. Biomarker‐anchored prospective cohorts targeting the empty cells should be the next research priority.

## 1 Introduction

Vegetables are a foundation of dietary recommendations worldwide, supplying fiber, polyphenols, and micronutrients with established gut‐microbiota and inflammatory‐barrier benefits (Wan et al. 2019). They are simultaneously the principal nonoccupational route by which residues of synthetic pesticides enter the human diet. Routine national monitoring programs include the European Food Safety Authority (EFSA) in the European Union, the US Food and Drug Administration Pesticide Residue Monitoring Program, and the Japanese Positive List System. These programs consistently detect quantifiable residues of organophosphates (OPs), pyrethroids, neonicotinoids, organochlorine (OC) breakdown products (DDT/DDE), and the herbicide glyphosate on conventionally produced leafy greens, cruciferous vegetables, root vegetables, and solanaceous fruits (European Food Safety Authority (EFSA) et al. 2025; occurrence by pesticide class and vegetable group is summarized in Table S1). These programs vary substantially in detection‐limit and reporting‐cycle, and vegetable‐specific residue patterns shift further with preparation state (whole vs. washed vs. peeled vs. cooked). Although individual residues typically fall below maximum residue limits, cumulative chronic dietary exposure to residue mixtures remains a population‐level concern, especially among pregnant women, infants, and children (National Research Council 1993; Temkin et al. 2025). These monitoring programs were moreover built around synthetic active ingredients. Natural and biopesticide residues used in organic and conventional production (e.g., pyrethrins, azadirachtin, rotenone, spinosad, copper, and *Bacillus thuringiensis*) and plant‐innate defensive metabolites intrinsic to many vegetables (e.g., glucosinolates and glycoalkaloids) lie largely outside routine multiresidue surveillance. This is an asymmetry we develop in Section 5.6. Advances in analytical sensitivity compound interpretation: modern gas‐ and liquid‐chromatography tandem mass‐spectrometry methods now quantify residues at concentrations previously undetectable (Anastassiades et al. 2003). A rise in detection frequency can therefore reflect improved instrumentation as much as increased exposure, and detection should be read alongside the reported limit of detection.

The past decade has produced a substantial mechanistic literature linking pesticide exposures to gut microbiota composition, intestinal barrier integrity, low‐grade inflammation, and functional and inflammatory GI diseases. Animal models, mostly mice fed chlorpyrifos, glyphosate, or pyrethroids at environmentally relevant doses, reproducibly show altered Bacteroidetes/Firmicutes ratio and reduced short‐chain fatty acid (SCFA) production (Liu et al. 2017; Ma et al. 2023). They also show increased intestinal permeability indexed by zonulin or lipopolysaccharide‐binding protein, and aggravated experimental colitis (Réquilé et al. 2018; Yang et al. 2020; Yuan et al. 2025). Yet two persistent disconnects exist. First, food‐science research and gut‐medicine research operate in noncommunicating literatures. Studies of residue‐removal efficiency through washing, peeling, and cooking rarely propagate into health‐outcome inference, and gut‐microbiota studies rarely back‐calculate the dietary exposure that produced their reported associations. Second, human‐population evidence on the residue–microbiota–GI axis is genuinely sparse. A PubMed precheck returned 207 hits across all five focal pesticide classes combined, of which only a small subset directly examines the chain from vegetable consumption through biomarker‐confirmed residue exposure to a microbiota, barrier, functional‐GI, or IBD outcome. These mechanistic effects depend on dose (at vs. above the acceptable daily intake [ADI]), exposure duration (subchronic vs. intergenerational), and life stage (in utero, infancy, adulthood). The Bacteroidetes/Firmicutes ratio they reference has no clinically validated reference value and varies with age, geography, diet, and sequencing method, so it is interpretable only as a within‐study relative comparison rather than against a universal norm.

The 2018‐onward proliferation of low‐cost 16S rRNA and shotgun metagenomic sequencing in human cohorts has been matched by mature urinary biomarker assays for OP metabolites (dialkyl phosphates, 3,5,6‐trichloro‐2‐pyridinol), pyrethroid metabolites (3‐phenoxybenzoic acid), and glyphosate (urinary glyphosate, AMPA). Together, these advances have now made systematic synthesis tractable. Recent contributions include the TwinsUK pesticide–microbiome study (Mesnage et al. 2022), a case–control study of pesticide exposure and irritable bowel syndrome (IBS) in an Andalusian agricultural community (Rueda‐Ruzafa et al. 2023), two large US cohorts on childhood and adult pesticide exposure with IBD incidence (Chen, Parks, et al. 2024; Chen, Woo, et al. 2024), and an observational analysis of ambient OP exposure and the human gut microbiome (Zhang et al. 2024). Pregnancy cohorts further show that pesticide × gut‐microbiota mediation underlies gestational outcomes (Dong et al. 2025; Yang et al. 2025). Several cluster‐randomized organic‐diet intervention trials with biomarker readouts add to this evidence (Abimbola et al. 2024; Bradman et al. 2015; Curl et al. 2019; Hyland et al. 2019).

We conducted this scoping review under the PRISMA extension for Scoping Reviews (PRISMA‐ScR) framework (Tricco et al. 2018). We defined the chain of interest as: vegetable‐derived pesticide residues (or biomarker proxies) → dietary exposure (FFQ paired with a residue database, duplicate diet, urinary biomarker confirmed against diet, or organic‐diet intervention) → measurable health endpoint within four prespecified outcome categories: gut microbiota composition and function; intestinal barrier integrity and inflammation; functional GI disorders (IBS, functional dyspepsia, celiac disease, prenatal‐exposure‐attributed diarrhea); and IBD (Crohn's disease, ulcerative colitis). GI cancers were excluded (covered by extensive standalone reviews). The review focused on five pesticide classes (OPs, pyrethroids, neonicotinoids, OC residues such as DDT/DDE/HCB, and glyphosate), with carbamates, phenoxy compounds, and triazines admitted only when comeasured alongside a focal‐class chemical. Animal and organoid mechanistic evidence is summarized in a tightly bounded mechanism brief in the discussion (Section 5.3) and does not enter the main extraction table. We had three objectives. The first was to map the human‐population evidence onto a pesticide‐class × outcome‐category gap matrix. The second was to synthesize the methodological landscape of vegetable–pesticide exposure assessment in epidemiology. The third was to identify food‐science to GI‐medicine bridge points that current evidence does not cross. Figure S1 summarizes the residue‐to‐outcome chain and the food‐science to gut‐medicine bridge that frame this review.

## 2 Methods

### 2.1 Reporting Framework and Protocol

We reported the review in conformance with PRISMA‐ScR (Tricco et al. 2018). The protocol (population/exposure/comparator/outcome [PECO] frame, eligibility criteria, search strategy, and extraction template) was finalized before screening and, together with all execution‐stage decisions, is available from the corresponding author on reasonable request. The completed PRISMA‐ScR checklist is provided in Table S4.

### 2.2 Eligibility Criteria

We included peer‐reviewed primary research published January 1, 2010 to the search date that satisfied all of: (i) **Population**, general human populations including pregnant women, mother–child pairs, infants, children, and adults, with exclusively occupational populations and acute‐poisoning case series excluded (mixed populations admitted when the analysis distinguished the dietary‐exposure component); (ii) **Exposure**, dietary pesticide exposure from vegetables captured by FFQ or 24‐h recall paired with a residue database, duplicate‐diet sampling, urinary or serum biomarkers paired with dietary attribution (urinary dialkyl phosphates for OPs, urinary 3‐phenoxybenzoic acid for pyrethroids, urinary glyphosate, serum *p,p*′‐DDE for OCs), or vegetable consumption frequency where local residue data supported the inference (biomarker‐only studies without dietary linkage were recorded as exposure‐assessment methodology references for Section 3.2 but not as outcome‐side rows; organic‐versus‐conventional dietary intervention trials were eligible when residue‐biomarker reductions were quantified); (iii) **Pesticide class**, at least one of OPs, pyrethroids, neonicotinoids, OC residues, or glyphosate; (iv) **Outcome**, gut microbiota (alpha or beta diversity, taxon abundance, functional gene/pathway), intestinal barrier and inflammation (zonulin, lipopolysaccharide‐binding protein, intestinal permeability, fecal calprotectin, serum C‐reactive protein when interpreted as a gut‐inflammation marker), functional GI (IBS by Rome II/III/IV criteria, functional dyspepsia, celiac disease, prenatal‐exposure‐attributed diarrhea), or IBD (Crohn's disease, ulcerative colitis); (v) **Design**, observational or interventional; (vi) **Language**, English peer‐reviewed only (Chinese‐language databases not searched). We excluded (a) GI cancers, (b) purely animal or in vitro/SHIME‐bioreactor studies (these populate the mechanism brief), (c) reviews (used only for citation tracing), (d) conference abstracts, and (e) nonpeer‐reviewed preprints. Per‐ and polyfluoroalkyl substances (PFAS), polybrominated diphenyl ethers, and polychlorinated biphenyls fall outside the five focal pesticide classes; where comeasured in a multipollutant panel they were retained only as in‐cohort comparators or negative controls and never as focal exposures, so evolving PFAS regulatory thresholds did not affect any inclusion or exclusion decision.

### 2.3 Information Sources and Search Strategy

We searched PubMed as the primary and only completed electronic database (2010 to present) and offset single‐database reliance with mandatory backward and forward citation tracing. The strategy combined three concept blocks with Boolean AND: (Block 1) pesticide‐class and chemical names plus relevant Medical Subject Headings (MeSH); (Block 2) vegetable and dietary‐source terms (vegetable*, leafy green, cruciferous, produce, FFQ, duplicate diet, organic diet, plus Vegetables[Mesh] and Diet[Mesh]); (Block 3) gut‐microbiota, barrier‐inflammation, functional‐GI, and IBD terms (gut microbiot*, 16S, metagenomic*, intestinal permeability, zonulin, fecal calprotectin, IBS, IBD, Crohn, ulcerative colitis, plus Gastrointestinal Microbiome[Mesh] and Inflammatory Bowel Diseases[Mesh]). Year limits 2010–present applied at the database level. The complete executed PubMed strategy, together with the protocol‐specified but unexecuted strings for Embase, Web of Science Core Collection, Scopus, and Cochrane CENTRAL, is provided in Table S3. The PubMed search returned 207 records at precheck and 263 unique records after the primary and supplementary queries and deduplication. Mandatory forward and backward citation tracing in Web of Science, Connected Papers, and Research Rabbit, on (a) all included reviews and (b) high‐citation primary studies, was performed as a required component of corpus completion. Gray‐literature sources included EFSA, the US Environmental Protection Agency Office of Pesticide Programs, the FAO/WHO Joint Meeting on Pesticide Residues, and the Chinese National Center for Food Safety Risk Assessment.

### 2.4 Selection, Extraction, and Risk‐of‐Bias

Records were deduplicated in EndNote then Zotero (Better BibTeX) and imported to Rayyan for single‐rater screening with a 10% double‐screened calibration check. The team member screened all titles and abstracts; the lead independently screened a 10% random sample, with Cohen's kappa ≥ 0.7 required before single‐rater full‐text screening proceeded. Extraction followed a predefined template, with one row per exposure × outcome combination. Pesticide‐class assignment used a controlled vocabulary. We used **ROBINS‐E** for observational studies and **Cochrane RoB 2.0** for randomized organic‐diet trials. For outcomes involving gut microbiota we additionally recorded a microbiome quality‐control rating at one of three levels: low (sequencing depth < 10,000 reads/sample, no batch‐effect reporting, or no rarefaction documentation), moderate, or high; any study failing two of the three criteria was graded low. The team member appraised all included studies; the lead appraised a 20% random sample. We recorded, for each study, whether exposure was ascertained by an analytically validated assay (gas‐ or liquid‐chromatography tandem mass spectrometry, or inductively coupled plasma mass spectrometry, for internal‐dose biomarkers) or by self‐report, recall, or ambient geographic proxy without internal‐dose measurement; this distinction is carried in Table 2 and in the ROBINS‐E exposure‐measurement domain. Because urinary metabolites (dialkyl phosphates, 3‐phenoxybenzoic acid, glyphosate, and AMPA) are short‐half‐life markers of recent internal dose excreted renally over hours to days, whereas lipophilic OCs in serum reflect years of bioaccumulation, each biomarker was interpreted as an exposure index on its own toxicokinetic timescale rather than against a clinical renal threshold. The agricultural source region, dietary pattern, and governing regulatory authority for each cohort are tabulated in Table 2, and the cross‐jurisdiction regulatory status with ADI or reference‐dose (RfD) anchors for the focal compounds is given in Table 3.

### 2.5 Synthesis

We did not pool effect estimates. Synthesis comprised (i) a narrative mapping across vegetable group, exposure‐assessment method, and outcome category (Section 3); (ii) a study‐density gap matrix indexed by pesticide class × outcome category, generated with a reproducible plotting script using the viridis color palette; (iii) a mechanism brief, capped at approximately 700 words and folded into Discussion Section 5.3; and (iv) a methodological‐challenges synthesis covering residue‐database localization, microbiome‐sequencing heterogeneity, and confounder‐set construction in dietary‐pesticide epidemiology.

## 3 Results: Mapping the Evidence

**FIGURE 1:**
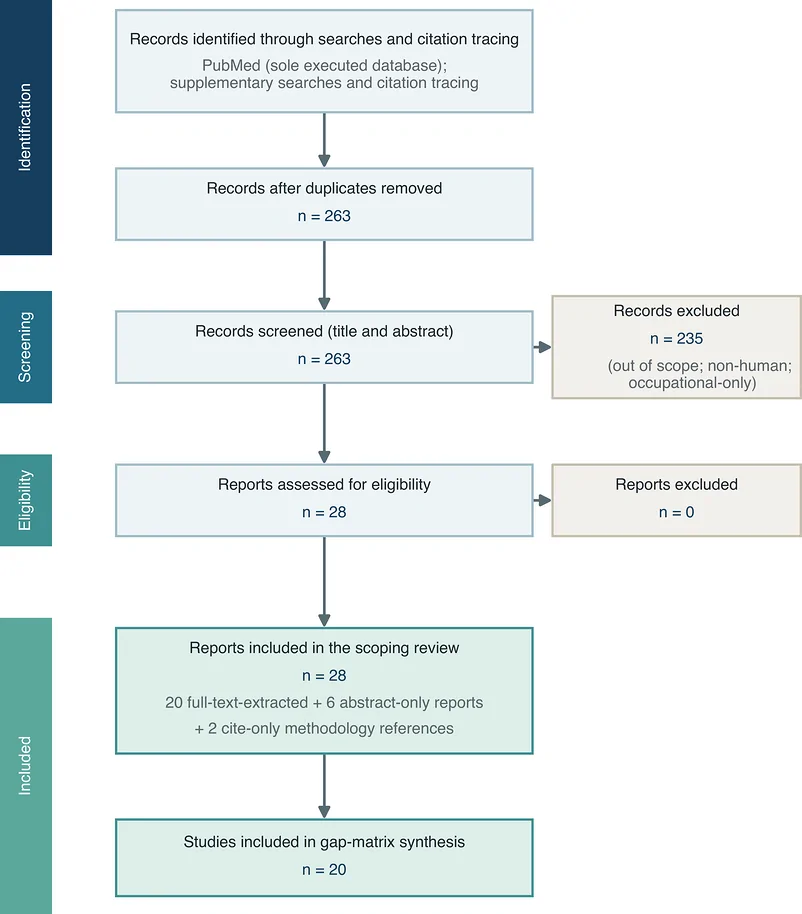
PRISMA‐ScR flow diagram of study selection. *Note*: The search and screening process identified 28 eligible reports, including 20 full‐text‐extracted studies used in the evidence synthesis.

Of the 28 included papers (Figure 1), 20 are full‐text‐extracted for the gap‐matrix synthesis, two are cite‐only methodology references (Bradman 2015 organic‐diet trial protocol; HELIX exposome design), and six are abstract‐only retentions cited in Section 1 motivation and Section 5.4 limitations (Lamichhane et al. 2026; Abimbola et al. 2024; Stanaway et al. 2023; Fagan et al. 2020; Curl et al. 2019; Hyland et al. 2019; primarily organic‐diet biomarker trials and limitations refs that do not measure GI outcomes themselves).

The 20 full‐text‐extracted human‐population studies span six study designs: large prospective cohorts of US women (Sister Study (Chen, Woo, et al. 2024), *n* = 48,382; Agricultural Health Study [AHS] applicators and spouses (Chen, Parks, et al. 2024), *n* = 68,480); a US community ambient‐exposure cohort (PEG, central California, Zhang et al. 2024, *n* = 190); a UK twin‐pair biomonitoring panel (TwinsUK, Mesnage et al. 2022, *n* = 65 pairs); an island‐population pooled birth‐and‐IBD‐registry study (Faroese CHEF, Hammer et al. 2019, *n* = 5698); a Spanish ecological case–control of IBS (Rueda‐Ruzafa et al. 2023); a Japanese small‐cohort biomarker‐and‐fecal‐SCFA study (Ueyama et al. 2022, *n* = 38); a Norwegian breast‐milk‐and‐infant‐microbiome cohort (NoMIC, Iszatt et al. 2019, *n* = 267 mother–child pairs); a multipollutant pregnancy exposome (INMA, Maitre et al. 2018, *n* = 750); a Yakima Valley farmworker oral‐microbiome cohort (Stanaway et al. 2017, *n* = 117); a South African indoor‐residual‐spraying birth cohort (VHEMBE, Davis et al. 2024, *n* = 629); three Romanian plant‐rich‐diet cohorts (Tomuța series: Tomuta, Gîtea, et al. 2026, *n* = 26 case series; Tomuta, Caltea, et al. 2026, *n* = 93; Tomuta et al. 2025 cohort sample size not reported); two nested Chinese pregnancy cohorts from the Maternal‐Child Cohort of Maternity and Child Health Hospital of Nanjing (MCMC; Dong et al. 2025, *n* = 731, gestational anemia outcome, and Yang et al. 2025, *n* = 852, gestational diabetes mellitus [GDM] outcome, same recruitment 2017–2018, same 29‐pesticide GC–MS/MS panel, same Nanjing Medical University team, sample overlap likely but unstated and these two papers are reported as one cohort with two outcomes for sample‐size accounting); a 53‐pollutant hospitalized‐infant gut microbiome study from Hunan, China (Xiang et al. 2025, *n* = 304 infants; population caveat: hospitalized neonatal‐ward infants with GI symptoms, 92.1% on antibiotics, not generalizable to healthy infants); a pilot birth‐cohort microbiome study from Rio de Janeiro, Brazil (PIPA pilot, Naspolini et al. 2021, *n* = 94 mother–infant dyads; pesticide signal smaller than comeasured metals/PFAS); a single‐site US pediatric celiac case–control pilot (NYU Hassenfeld, Gaylord et al. 2020, *n* = 88; 30 cases + 58 controls); and a Mexican boys‐only birth cohort of in utero DDT exposure and infant diarrhea (Tapachula, Chiapas, Cupul‐Uicab et al. 2017, *n* = 747 boys). Combined sample size now exceeds 125,000 across designs, dominated by the AHS (Chen, Parks, et al. 2024) and Sister Study (Chen, Woo, et al. 2024); the two MCMC nested cohorts (Dong et al. 2025; Yang et al. 2025) are counted once for cohort‐level sample‐size accounting (∼852, taking the larger of the two analytic samples) given likely overlap.

### 3.1 Vegetable‐Source Residue Spectrum

The gap matrix contains 20 nonempty cells out of 60 canonical pesticide‐class × outcome combinations (33%). Following the scoping‐review evidence‐density convention, multiclass biomonitoring panels contribute to every cell their assayed pesticide classes cover and are deduplicated only at the total‐N level. Throughout the Results, a column *n* denotes the number of distinct papers contributing to that outcome column, whereas Table 1 cell and column totals count class‐level study‐contributions; because a multiclass panel contributes to several pesticide‐class rows, Table 1 column totals exceed the distinct‐paper counts. The classes populating those cells (Table 1) are OCs, OPs, pyrethroids, glyphosate, triazine, chloroacetamide, conazole, herbicides, mixed/unspecified, carbamate, and fungicide, the latter two appearing in a single IBD cell each from the AHS (Chen, Parks, et al. 2024); neonicotinoid, phenoxy, and bromine‐containing rows remain empty across all four outcome categories.

**TABLE 1:** Evidence density across pesticide classes and gastrointestinal outcomes.

Pesticide class	Microbiota	Barrier and inflammation	Functional GI/IBS/celiac	IBD	Total
Organochlorine (OC)	9 ±	4 ±	4 +	3 +	**20**
Organophosphate (OP)	11 ±	2 ±	1 +	1 +	**15**
Pyrethroid	7 ±	3 ±	2 +	1 +	**13**
Glyphosate	5 ±	2 ±	1 +	—	**8**
Triazine	2 ±	—	—	—	**2**
Mixed/unspecified	3 ±	—	—	—	**3**
Carbamate	—	—	—	1 +	**1**
Fungicide	—	—	—	1 +	**1**
Neonicotinoid	—	—	—	—	**0**
Phenoxy	—	—	—	—	**0**
**Total (study‐contributions)**	**37**	**11**	**8**	**7**	**63**

*Note*: Counts represent study contributions within each pesticide‐class × outcome category. Multiclass biomonitoring studies were counted for each relevant pesticide class. Effect direction is summarized as + positive, − inverse, ± mixed, and — no data. The four columns collapse six prespecified outcome subcategories; under the 60‐cell canonical matrix (10 pesticide classes × 6 subcategories), 20 cells (33%) are populated.

Study‐density counts for each pesticide class and outcome category are provided in Table 1. OCs and OPs were the most broadly represented classes, each populating all four outcome categories, and the gut‐microbiota column was the densest. Pyrethroids likewise spanned all four outcome categories, whereas glyphosate reached three (microbiota, barrier and inflammation, and functional GI) but contributed no inflammatory bowel disease evidence. Triazine and a mixed/unspecified class appeared in the microbiota column only, through the multipesticide MCMC pregnancy panels (Dong et al. 2025; Yang et al. 2025), in which a single environmental risk score (ERS) rather than per‐class coefficients was the principal exposure measure. Chloroacetamide, conazole, and herbicide chemicals were measured only alongside focal‐class compounds (Dong et al. 2025; Yang et al. 2025; Xiang et al. 2025) and are folded into the OC and OP rows of the matrix; carbamate and fungicide each contributed a single inflammatory bowel disease cell from the AHS (Chen, Parks, et al. 2024).

Residue‐attribution methodology splits into four styles. (i) **Biomarker‐confirmed dietary attribution** is represented by the TwinsUK pesticide‐biomonitoring panel (Mesnage et al. 2022; urinary dialkyl phosphates, 3‐phenoxybenzoic acid, glyphosate, AMPA, and 4‐bromo‐2‐chlorophenoxyacetic acid [Br2CA] in 65 pairs, FFQ‐paired) and the Japanese SCFA cohort (Ueyama et al. 2022, urinary dialkyl phosphates, 3‐phenoxybenzoic acid, and glyphosate in 38 older adults, vegetable‐intake covariate). (ii) **Self‐reported pesticide use without biomarker** dominates the AHS (Chen, Parks, et al. 2024, ever‐use of 50 pesticides recalled at 1993–1997 enrolment) and Sister Study (Chen, Woo, et al. 2024, childhood and adolescent residential‐pesticide use recalled by adult women). (iii) **Ambient or ecological exposure proxies** include the PEG geographic information system (GIS)‐based pesticide‐application records linked to residence (Zhang et al. 2024), the Andalusian agronomic high‐versus‐low pesticide‐use district contrast (Rueda‐Ruzafa et al. 2023), and the Faroese diet‐based persistent‐organic‐pollutant burden ascertainment (Hammer et al. 2019). (iv) The Romanian Tomuța series (Tomuta et al. 2025; Tomuta, Caltea, et al. 2026; Tomuta, Gîtea, et al. 2026) introduces a fourth methodological style: **dietary‐modeling estimation of residue intake from national‐surveillance residue data combined with individual food‐frequency records**, deployed against urinary metabolomic outcomes rather than direct microbiome sequencing in two of the three papers.

### 3.2 Exposure‐Assessment Methodology

Three methodological observations matter for the discussion. First, the TwinsUK study (Mesnage et al. 2022) is the only full‐text‐extracted paper that combines FFQ + biomarker + shotgun metagenomics in a single design: it reports OP and pyrethroid metabolite detection in 100% of urine samples and glyphosate detection in 53%, with FFQ‐confirmed positive association between fruit‐and‐vegetable consumption and OP‐metabolite excretion. Second, the Japanese SCFA cohort (Ueyama et al. 2022) operationalizes a tractable lower bound design (small *n* = 38, targeted urinary biomarkers paired with targeted fecal SCFA chemistry, controlling for vegetable intake), yielding a significant inverse multiple‐regression coefficient between urinary dialkyl phosphate excretion and fecal acetate (*β* = −24.0, *p* < 0.001; adjusted *R* ^2^ = 0.751). Third, organic‐diet intervention trials retained for Section 3.2 cite‐only (Bradman et al. (2015), *n* = 40 children pre‐/postorganic‐intervention urinary dialkyl phosphate reduction; HELIX exposome multicohort exposure‐mapping (Papadopoulou et al. 2019), *n* = 818 pregnant women) establish biomarker‐reduction methodology even though they do not measure GI outcomes themselves.

Six biomarker‐confirmed cohorts warrant explicit population description. The MCMC nested pregnancy cohort in Nanjing, China (Dong et al. 2025, *n* = 731 analytic from 1527 enrolled, gestational anemia outcome; Yang et al. 2025, *n* = 852 with a 506‐subject shotgun metagenomic subset, GDM outcome) used maternal serum collected at 22–24 weeks’ gestation analyzed by GC–MS/MS triple‐quadrupole for 29 pesticides spanning OC, OP, triazine, chloroacetamide, and conazole classes, with limits of detection 0.000675–2.14 ng/mL. The Hunan, China hospitalized‐infant cohort (Xiang et al. 2025, *n* = 304 infants from a neonatal ward at Hunan Children's Hospital) measured a 53‐pollutant infant‐plasma panel (14 herbicides, 9 OCPs, 8 OPs, 7 PAHs, 6 PBDEs, 6 PCBs, 3 pyrethroids), with the population caveat that 92.1% of infants had received antibiotics and 64.5% had pneumonia at recruitment. The PIPA pilot birth cohort in Rio de Janeiro, Brazil (Naspolini et al. 2021, *n* = 94 mother–infant dyads from 142 enrolled at the public maternity clinic) measured 49 chemicals across maternal blood, urine, and cord blood by ICP‐MS and LC–MS/MS, including DDT/DDE and pyrethroid metabolites 3‐PBA and 4‐FPBA; > 50% of DDT and pyrethroid measurements fell below the limit of detection, restricting analyses to categorical (exposed vs. nonexposed) form. The NYU Hassenfeld pediatric celiac case–control (Gaylord et al. 2020, *n* = 88: 30 cases, 58 controls from a single‐site outpatient pediatric GI clinic, predominantly non‐Hispanic white) measured serum POPs (PBDEs, DDE only, not DDT, and PFAS) by the Kannan Lab. The Tapachula boys‐only birth cohort in Chiapas, Mexico (Cupul‐Uicab et al. 2017, *n* = 747 boys from 870 enrolled, originally designed to test the DDT androgen‐blocking hypothesis) measured maternal serum DDE and DDT collected within 1 day of delivery by GC–MS, with median DDE 2.7 µg/g lipid (approximately 13 × US NHANES 2003–2004 women).

The four full‐text organic‐diet intervention trials now in our corpus permit a sharper comparison of biomarker‐reduction efficacy across study designs. Bradman et al. (2015), a 6‐day crossover in 40 California children, reported median urinary dialkyl‐phosphate reductions of approximately 40% on the organic phase. Curl et al. (2019); CC‐PEACE, *n* = 23 pregnant women; cohort details in Methods) reported a 24‐week organic‐produce intervention reducing urinary 3‐PBA but not OP metabolites in a low‐baseline population. Hyland et al. (2019), a 6‐day organic‐diet pilot in US children and adults (*n* = 16), reported 60%–95% reductions in urinary glyphosate and AMPA. Abimbola et al. (2024), the only intervention in our corpus that measured an inflammation‐related secondary outcome, reported reductions in urinary OP metabolites and a parallel decrease in serum CRP in children participating in a Cyprus organic‐diet cluster‐randomized crossover trial. The Fagan et al. (2020) substudy on glyphosate specifically reported urinary glyphosate reductions of approximately 70% over a 6‐day organic intervention. Across these five intervention designs, biomarker‐reduction efficacy ranges from approximately 40% (OP metabolites, short‐window children) to 95% (glyphosate, short‐window adults), with intervention duration and baseline exposure intensity as the primary drivers; only Abimbola et al. (2024) measured a downstream inflammation marker, and even that single design did not measure microbiota composition.

The biomarker‐versus‐recall trade‐off is reflected in the ROBINS‐E appraisal, which flagged recall‐based and biomarker‐confirmed exposure designs as serious‐risk and moderate‐risk in the exposure‐measurement domain, respectively. The biomarker‐confirmed cohorts now span seven designs with gestational and infant biomarker measurement (TwinsUK (Mesnage et al. 2022), *n* = 65 pairs; NoMIC (Iszatt et al. 2019), *n* = 267; Japanese SCFA (Ueyama et al. 2022), *n* = 38; MCMC pregnancy cohorts (Dong et al. 2025; Yang et al. 2025), *n* = 852; PIPA Brazilian pilot (Naspolini et al. 2021), *n* = 94; Tapachula Mexico (Cupul‐Uicab et al. 2017), *n* = 747; NYU pediatric celiac (Gaylord et al. 2020), *n* = 88), trading sample size for exposure‐assessment quality.

### 3.3 Outcome Evidence by Category

Table 2 summarizes all 20 full‐text‐extracted studies, including a critical appraisal of whether each study's finding supports its stated hypothesis. Of the 20, 13 support their hypothesis within their design, five are partial (the finding partly supports the hypothesis, or the focal‐pesticide signal is secondary or imprecise), one does not support it (the analyzed contrast carried no pesticide‐exposure variation), and one is overall null with a positive subgroup. A recurring pattern, developed in the subsection syntheses below and in Section 4, is that apparent direction heterogeneity within an outcome column tracks the exposure‐assessment regime (biomarker‐confirmed vs. ambient‐proxy vs. recall‐based) more than it reflects divergent biology.

**TABLE 2:** Characteristics and appraisal of included human studies.

**Study (cohort)**	**Country**	**Design; population (n)**	**Pesticide class; exposure assessment**	**Outcome category**	**Key result (95% CI)**	RoB*	**Finding vs. stated hypothesis**
Tomuța 2026 (Oradea convenience cohort)	Romania	Cross‐sectional case series; plant‐rich‐diet adults (*n* = 26)	Multiclass (OP, OC, pyrethroid, glyphosate); estimated dietary intake (FFQ + national residue modeling), no biomarker	Microbiota; barrier/inflammation	Descriptive only (no regression): reproducible urinary organic‐acid and dysbiosis/oxidative‐stress pattern in 100% of high‐intake participants	Serious	Supports (descriptive; no comparator or exposure contrast)
Tomuța 2026 (Plant‐Rich‐Diet GI‐symptoms cohort)	Romania	Prospective cohort; plant‐rich‐diet adults with GI symptoms (*n* = 93; metabolomics subset *n* = 50)	Multiclass background (OP, OC, pyrethroid, glyphosate); uniform across strata, no contrast	Microbiota	Lower physical‐activity strata had reduced Shannon diversity; estimated pesticide intake did not differ between strata	Moderate	Does not support (physical activity, not pesticide, drove the difference; exposure uniform)
Dong 2025 (MCMC, Nanjing)	China	Prospective pregnancy cohort (*n* = 731)	29‐compound serum panel (OP, OC, triazine, chloroacetamide, conazole); GC–MS/MS biomarker + ERS	Microbiota (gestational anemia)	Furalaxyl × anemia OR 4.19 (1.05, 16.41); Shannon diversity mediates ∼9% of pesticide → RBC path	Moderate	Supports (biomarker‐confirmed; microbiota‐mediated)
Tomuța 2025 (Plant‐Based‐Diet cohort, Oradea)	Romania	Cross‐sectional; plant‐based‐diet adults (n not reported)	OP (chlorpyrifos, diazinon frequently tested); all food samples < MRL	Microbiota	Neuroactive dysbiosis (low *Bifidobacterium*/*Lactobacillus*, high *Oscillibacter*/*Alistipes*) + raised proinflammatory markers despite MRL‐compliant food	Serious	Supports the “clean‐eating paradox” hypothesis (but unidentifiable: no within‐cohort exposure variation)
Xiang 2025 (Hunan Children's Hospital)	China	Cross‐sectional, hospitalized neonatal‐ward infants (*n* = 304; 92% on antibiotics)	53‐pollutant plasma panel (OC, OP, pyrethroid, herbicide); GC–MS/MS biomarker, BKMR	Microbiota	Phosalone × Bifidobacterium −0.620; *o*,*p*′‐DDT × δ‐HCH synergistic interaction on *Pseudomonas* (*p* = 0.020)	High	Supports (hypothesis‐generating only; heavy clinical/antibiotic confounding)
Yang 2025 (MCMC, Nanjing)	China	Prospective pregnancy cohort (*n* = 852; metagenomic subset 506)	29‐compound serum panel; GC–MS/MS biomarker + elastic‐net ERS	Microbiota (gestational diabetes)	ERS adjusted OR 4.5 (1.4, 13.8); Prevotella‐low subgroup ∼10‐fold risk; *Dorea*‐branch mediation; internal‐validation AUC 0.83	Moderate	Supports (strongest biomarker‐confirmed quantitative signal in corpus)
Chen 2024 (Sister Study)	USA	Prospective cohort, adult women (*n* = 48,382)	OC (DDT‐era residential/farm); recalled self‐report, no biomarker	IBD	DDT‐era residence (> 6 × vs. never) HR 1.56 (1.06, 2.31); farm‐application HR 1.85–2.58 (CIs cross 1)	Moderate	Supports (primary DDT‐era exposure significant; several subgroups null)
Chen 2024 (Agricultural Health Study)	USA	Prospective cohort, applicators + spouses (*n* = 68,480)	Multiclass 50‐pesticide panel (OC, OP, pyrethroid, carbamate, herbicide, fungicide); ever‐use self‐report	IBD	Dieldrin HR 1.59 (1.03, 2.44); toxaphene 1.61 (1.17, 2.21); terbufos ever‐use 1.53 (1.19, 1.96); tertiles elevated without monotonic gradient	Moderate	Supports (multiple OC/OP signals significant; threshold‐type dose‐response)
Davis 2024 (VHEMBE)	South Africa	Birth cohort, mother–child pairs (*n* = 629; children 3.5–5 y)	OC (DDT/DDE) + pyrethroid; serum + urinary biomarker (indoor residual spraying)	Functional GI; barrier/inflammation	Pyrethroid cis‐DBCA per 10‐fold → ear‐infection IRR 1.4 (1.0, 2.1); persistent‐diarrhea IRR positive (value in full text)	Moderate	Partial (ear‐infection borderline; diarrhea signal present but imprecise)
Zhang 2024 (PEG, California)	USA	Community cohort, older adults (*n* = 190)	OP; ambient GIS application proxy (500 m, 5‐y), no biomarker	Microbiota	22 differentially abundant genera + 34 perturbed MetaCyc pathways (reduced B‐vitamin biosynthesis); no single direction	Moderate	Supports (composition altered; direction mixed; ambient proxy)
Rueda‐Ruzafa 2023 (Andalusia)	Spain	Population‐based ecological case–control (n not reported)	Multiclass (OP, pyrethroid, glyphosate, OC); high‐ vs. low‐use district (ecological proxy)	Functional GI (IBS); microbiota	IBS unadjusted OR 1.16 (1.13, 1.20); females 2.62 (2.54, 2.70); males 1.33 (1.26, 1.41)	Serious	Supports (ecological design; strong female‐specific signal)
Mesnage 2022 (TwinsUK)	UK	Twin‐pair biomonitoring panel (*n* = 65 pairs)	OP, pyrethroid, glyphosate; urinary biomarker + FFQ + shotgun metagenomics	Microbiota	OP/pyrethroid/glyphosate detected in 100%/53%/53%; glyphosate excretion positively associated with fecal species richness; 34 metabolite associations (FDR < 0.05)	Low‐Moderate	Partial (direction contrary to simple “pesticide harms microbiome” prior)
Ueyama 2022 (Nagoya)	Japan	Cross‐sectional, healthy older adults (*n* = 38)	OP, pyrethroid, glyphosate; urinary biomarker + fecal SCFA	Microbiota; barrier/inflammation	Urinary DAP × fecal acetate *β* = −24.0 (*p* < 0.001; adj *R* ^2^ = 0.751); DAP × lactate *r* = −0.391	Moderate	Supports (clean inverse OP → SCFA signal, vegetable‐intake adjusted)
Naspolini 2021 (PIPA, Rio de Janeiro)	Brazil	Birth cohort, mother–infant dyads (*n* = 94)	OC (DDT/DDE) + pyrethroid; multimatrix biomarker (> 50% < LOD)	Microbiota	DDT (exposed vs. non) → Lachnospiraceae ↑ meconium, Megasphaera ↓ 3‐mo; metal effects (Pb *β* = 0.248) larger than pesticide	Moderate	Partial (pesticide signal real but secondary to comeasured metals/PFAS)
Gaylord 2020 (NYU Hassenfeld)	USA	Single‐site case–control pilot, children (*n* = 88; DDE subset 81)	OC (DDE only; PBDE/PFAS are nonpesticide POPs); serum biomarker	Functional GI (celiac)	DDE × celiac adjusted OR 2.04 (1.08, 3.84); females 13.0 (1.54, 110) [very wide]	Serious	Supports (first POP × celiac signal; pilot, unstable subgroup CIs, collider risk)
Hammer 2019 (Faroese CHEF)	Faroe Islands	Cohort, mother–child (*n* = 5698; 37 IBD cases)	OC (DDT/DDE/PCBs); serum + breast‐milk POP, dietary‐vehicle ascertainment	IBD	*p*,*p*′‐DDT high vs. low RR 3.04 (1.12, 8.30); pilot‐whale RR 1.02 and fish RR 1.01 (null)	Serious	Supports (chemical signal positive; dietary‐vehicle null; few cases, wide CI)
Iszatt 2019 (NoMIC)	Norway	Birth cohort, mother–infant pairs (*n* = 267)	OC (HCB, DDE, DDT, HCH) + PCB/PBDE/PFAS; breast‐milk biomarker	Microbiota; barrier/inflammation	Per‐SD PBDE‐28 → propionic acid −24% (−35, −14); PCB‐209 → acetic −15%; PFOA → propionic +61% (nonpesticide)	Low–Moderate	Partial (strongest SCFA effects from nonpesticide POPs; focal OC signal weaker)
Maitre 2018 (INMA)	Spain	Prospective pregnancy cohort (*n* = 750)	OC (within POP exposome panel); serum biomarker, ^1^H‐NMR urinary metabolome	Barrier/inflammation; microbiota	Sparse but systematic exposome associations (1495 tests); p,p′‐DDE × specific metabolites significant; strongest signal thallium (*ρ* = −0.28)	Low–Moderate	Partial (method demonstration; OC‐pesticide associations sparse; strongest signal nonpesticide)
Cupul‐Uicab 2017 (Tapachula)	Mexico	Boys‐only birth cohort (*n* = 747; median DDE ∼13× US NHANES)	OC (DDT + DDE); maternal serum biomarker (GC–MS)	Functional GI (infant diarrhea)	Overall DDE high vs. low aIRR 1.14 (0.94, 1.39) null; urban boys aIRR 1.39 (1.07, 1.80); rural null	Moderate	Null overall (urban subgroup positive; authors invoke rural coexposure masking)
Stanaway 2017 (Yakima Valley)	USA	Cohort, farmworkers + nonfarmworkers (*n* = 117)	OP (azinphos‐methyl); blood biomarker, seasonal contrast	Microbiota (oral)	Reduced oral Streptococcus and overall diversity in azinphos‐methyl‐positive subjects (FDR *q* < 0.05; seven taxa)	Moderate	Supports (clear seasonal exposure → oral‐microbiome alteration)

*Note*: This table summarizes study design, exposure assessment, outcomes, main findings, risk of bias, and whether the findings supported each study's stated hypothesis. RoB was assessed using ROBINS‐E or Cochrane RoB 2.0. Verdict definitions: Supports = the study's primary finding is in the hypothesized direction and statistically supported within its design; Partial = the finding partly supports the hypothesis or the focal‐pesticide signal is secondary or imprecise; Does not support = the hypothesized exposure was not the driver; Null overall = no overall association, with any subgroup signal noted. Effect estimates are reproduced verbatim from each source.

Abbreviations: aIRR, adjusted incidence rate ratio; AUC, area under the receiver‐operating‐characteristic curve; BKMR, Bayesian kernel machine regression; CHEF, Children's Health and the Environment in the Faroes cohort; CI, confidence interval; cis‐DBCA, cis‐3‐(2,2‐dibromovinyl)‐2,2‐dimethylcyclopropane carboxylic acid; DAP, dialkyl phosphate; DDE, dichlorodiphenyldichloroethylene; DDT, dichlorodiphenyltrichloroethane; ERS, environmental risk score; FDR, false‐discovery rate; FFQ, food‐frequency questionnaire; GC–MS/MS, gas chromatography–tandem mass spectrometry; GI, gastrointestinal; GIS, geographic information system; HCB, hexachlorobenzene; HCH, hexachlorocyclohexane; HR, hazard ratio; IBD, inflammatory bowel disease; IBS, irritable bowel syndrome; INMA, Infancia y Medio Ambiente cohort; IRR, incidence rate ratio; LOD, limit of detection; MCMC, Maternal–Child Cohort of the Maternity and Child Health Hospital of Nanjing; MetaCyc, Metabolic Pathway Database; MRL, maximum residue level; NMR, nuclear magnetic resonance; NoMIC, Norwegian Microbiota Cohort; OC, organochlorine; OP, organophosphate; OR, odds ratio; PBDE, polybrominated diphenyl ether; PCB, polychlorinated biphenyl; PEG, Parkinson's, Environment, and Genes study; PFAS, per‐ and polyfluoroalkyl substances; PIPA, Programa Infância e Poluentes Ambientais cohort; POP, persistent organic pollutant; RBC, red blood cell; RoB, risk of bias; RR, relative risk; SCFA, short‐chain fatty acid; SD, standard deviation; TwinsUK, United Kingdom Adult Twin Registry cohort; VHEMBE, Venda Health Examination of Mothers, Babies and their Environment cohort.

#### 3.3.1 Gut Microbiota (the Densest Column, 14 Distinct Papers Contributing 37 Study‐Contributions Across Multiple Class‐Rows After Re‐Extraction)

OP‐class evidence dominates: ambient‐OP × 16S microbiome in central California (Zhang et al. 2024), urinary‐DAP × fecal SCFA in Japan (Ueyama et al. 2022), urinary‐DAP × shotgun metagenomics in TwinsUK (Mesnage et al. 2022), the Yakima Valley farmworker oral‐microbiome study (Stanaway et al. 2017, blood azinphos‐methyl detection × oral *Streptococcus* reduction), the Andalusian ambient‐pesticide × IBS‐with‐microbiome‐context analysis (Rueda‐Ruzafa et al. 2023), the three Romanian Tomuța papers (Tomuta et al. 2025; Tomuta, Caltea, et al. 2026; Tomuta, Gîtea, et al. 2026), and three newly extracted Chinese pregnancy‐and‐infant cohorts (Dong et al. 2025; Yang et al. 2025; Xiang et al. 2025). OC, pyrethroid, glyphosate, triazine, and mixed/unspecified classes also contribute to this column, including the OC‐anchored cohorts Naspolini et al. 2021 (PIPA), Iszatt et al. 2019 (NoMIC), and Maitre et al. 2018 (INMA; Table 1). The most quantitatively precise effect estimate in this column is the Japanese DAP‐acetate *β* = −24.0 (*p* < 0.001) in Ueyama et al. (2022). The Mesnage TwinsUK study (Mesnage et al. 2022) reports a positive Spearman rank correlation between urinary glyphosate excretion and overall fecal bacterial species richness in shotgun metagenomic data, contrasting with the Japanese DAP‐SCFA inverse pattern; this is direction heterogeneity within this column that the gap‐matrix mixed summary preserves. The MCMC GDM analysis (Yang et al. 2025) provides the strongest newly added quantitative anchor in this column: a multipesticide ERS constructed by elastic net regression yielded an adjusted odds ratio (OR) of 4.5 (95% confidence interval [CI], 1.4, 13.8; *p* = 0.008) for GDM, with a Prevotella‐low microbiota subgroup carrying an approximately 10‐fold susceptibility, a *Dorea*‐branch microbiota mediating part of the pesticide‐to‐GDM path, and an integrated predictive model achieving an internal‐validation AUC of 0.83 (95% CI, 0.75, 0.92), among the strongest reported OP × pregnancy‐outcome quantitative findings in our corpus. These estimates come from a single cohort with a wide CI and an internally validated elastic‐net model without external validation, so their apparent precision should not be overinterpreted. The companion MCMC analysis (Dong et al. 2025) reports a furalaxyl × gestational‐anemia second‐trimester OR of 4.19 (95% CI, 1.05, 16.41) and a quantitative microbiota‐mediated path estimate (∼9% mediation share via Shannon diversity) of a pesticide → maternal‐hematological path. The Hunan hospitalized‐infant 53‐pollutant analysis (Xiang et al. 2025) reports a synergistic *o*,*p*′‐DDT × δ‐HCH interaction on *Pseudomonas* overgrowth (interaction *p* = 0.020) using Bayesian Kernel Machine Regression, with the important caveat that the population is hospitalized neonatal‐ward infants with 92.1% antibiotic exposure and that findings should be interpreted as hypothesis‐generating rather than as evidence for healthy‐infant exposure response.

##### 3.3.1.1 Synthesis

Across the 14 microbiota‐contributing studies the majority report a pesticide‐associated perturbation, but the direction is not uniform and tracks exposure‐assessment regime rather than biology: biomarker‐confirmed functional readouts converge on suppression of SCFA‐producing function (urinary dialkyl phosphate to fecal acetate, beta = −24.0 in Ueyama et al. 2022); biomarker‐confirmed compositional readouts diverge (glyphosate excretion to increased fecal species richness in TwinsUK, Mesnage et al. 2022, contrary to a simple harm prior); ambient‐proxy designs show taxon‐level shifts without diversity loss (Zhang et al. 2024); and the multiclass serum‐panel pregnancy cohorts report adverse, microbiota‐mediated clinical associations (Dong et al. 2025; Yang et al. 2025). The one study in which pesticide exposure did not vary across the analyzed contrast found physical activity, not pesticide burden, to drive diversity (Tomuta, Caltea, et al. 2026), underscoring that an interpretable association requires within‐cohort exposure variation. Read as a single direction the cell is mixed; read as four subquestions (biomarker by functional readout, biomarker by compositional readout, biomarker by clinical outcome with microbial mediation, and ambient proxy by compositional readout) the column is internally coherent and consistently supports a perturbation hypothesis while not supporting any single taxonomic signature (Table 2).

#### 3.3.2 Intestinal Barrier and Inflammation (*n* = 11 Study‐Contributions Across Five Distinct Papers)

The OC × barrier and inflammation cell is the densest (*n* = 4). The NoMIC breast‐milk‐toxicants study (Iszatt et al. 2019) provides the cleanest quantitative effect: per‐standard‐deviation polybrominated diphenyl ether 28 (PBDE‐28) in breast milk → infant fecal propionic acid −24% (95% CI, −35, −14); polychlorinated biphenyl 209 (PCB‐209) → acetic acid −15% (95% CI, −29, −0.4); perfluorooctanoic acid (PFOA) → propionic acid +61% (95% CI, 35, 87). PFOA is a nonpesticide persistent organic pollutant measured in the same NoMIC multipollutant panel and serves as an in‐cohort negative control for the OC‐pesticide signal; we discuss its role explicitly in Section 5.2. The VHEMBE pyrethroid indoor‐residual‐spraying study (Davis et al. 2024) reports per‐10‐fold cis‐DBCA increase → child ear‐infection incidence rate ratio (IRR) 1.4 (95% CI, 1.0, 2.1). Nonmicrobiome inflammation outcomes (CRP, calprotectin) are sparsely populated; the Cyprus organic‐diet trial (Abimbola et al. 2024) is the only intervention in our corpus that reports a parallel reduction in serum CRP alongside urinary OP‐metabolite reduction. Ear infection (otitis media) is a mucosal‐immunity readout adjacent to, rather than within, the intestinal barrier; we retain the VHEMBE contribution because the same cohort also reports a within‐scope persistent‐diarrhea (functional‐GI) outcome, and we note that disregarding the ear‐infection contribution would reduce the OC and pyrethroid barrier‐and‐inflammation cells by one contribution each without emptying any cell or altering the 20‐of‐60 (33%) density.

##### 3.3.2.1 Synthesis

This column is sparse, and its quantitatively strongest effects, the breast‐milk persistent‐organic‐pollutant to infant SCFA shifts in NoMIC (Iszatt et al. 2019), are driven mainly by comeasured nonpesticide pollutants (PBDE‐28, PCB‐209, PFOA), with the focal OC‐pesticide signal weaker; the pyrethroid mucosal‐immunity association (VHEMBE, Davis et al. 2024) is borderline (IRR 1.4, 95% CI, 1.0, 2.1). The evidence is therefore directionally suggestive of a barrier and SCFA effect but only partially supportive for the focal pesticide classes specifically (Table 2). Standard C‐reactive protein is moreover a nonspecific acute‐phase reactant that responds to exercise, minor injury, and transient stimuli, and transient postprandial inflammation can elevate nonfasting inflammatory markers independently of chronic exposure; high‐sensitivity CRP is the appropriate marker for the low‐grade systemic inflammation relevant to gut‐barrier dysfunction, and gut‐specific markers (fecal calprotectin, zonulin) are more specific still, so the single CRP‐based intervention signal here (Abimbola et al. 2024) should be read with that limitation.

#### 3.3.3 Functional GI/IBS/Celiac/Diarrhea (*n* = 8 Study‐Contributions Across Four Distinct Papers, Mostly Positive Direction After Re‐Extraction; Up From *n* = 4 in the Preliminary Count, With the OC × Functional‐GI Cell Rising From *n* = 2 to *n* = 3 and a Newly Populated OC × Functional‐GI (Celiac) Subcell at *n* = 1)

The Andalusian case–control (Rueda‐Ruzafa et al. 2023) is the principal evidence here. Total population unadjusted OR for IBS in high‐pesticide‐use districts was 1.16 (95% CI, 1.13, 1.20); the female‐specific crude OR was 2.62 (95% CI, 2.54, 2.70) and the male‐specific crude OR was 1.33 (95% CI, 1.26, 1.41). The published multivariable‐adjusted total‐population OR was 1.26, but the printed 95% CI as it appears in the source paper (1.12, 1.19) contains an upper bound below the point estimate and is therefore mathematically incoherent; we cite the unadjusted total‐population OR 1.16 (95% CI, 1.13, 1.20) as the primary effect estimate from this study and report the adjusted OR as 1.26 (printed 95% CI, 1.12, 1.19 as it appears in the source paper, retained verbatim despite the mathematical inconsistency) for transparency.

The full‐text retrieval of two previously abstract‐only papers in this category materially changes the cell composition. Gaylord et al. (2020) is among the earliest available evidence of a POP × pediatric celiac association: a single‐site case–control pilot at the NYU Hassenfeld Children's Hospital outpatient GI clinic (*n* = 88: 30 cases, 58 controls; 81‐subject DDE/PBDE subset of 28 cases and 53 controls) reports an adjusted DDE × celiac OR of 2.04 (95% CI, 1.08, 3.84) overall, with a sex‐stratified female adjusted OR of 13.0 (95% CI, 1.54, 110); the wide female CI reflects subgroup n in the low double digits and is properly read as an exploratory pilot signal. Critically, of the chemicals measured in Gaylord et al. (2020), PBDEs (flame retardants), DDE (the only pesticide‐derived chemical), and PFAS, only DDE falls within our pesticide‐class focal scope, and the earlier incidental description of Gaylord et al. (2020) as a “pyrethroid + OC” study has been corrected to OC (DDE) only. Cupul‐Uicab et al. (2017), the Tapachula boys‐only birth cohort (*n* = 747 boys, originally designed to test a DDT androgen‐blocking hypothesis), reports an overall null adjusted IRR of 1.14 (95% CI, 0.94, 1.39) for highest‐versus‐lowest maternal serum DDE on infant diarrhea; the positive signal is stratified to urban boys (adjusted IRR 1.39, 95% CI, 1.07, 1.80), with rural participants null. The authors hypothesize that the rural null reflects unmeasured pesticide coexposure, pyrethroids and carbamates from malaria‐control and agricultural use, that masks the underlying DDT/DDE effect (Cupul‐Uicab et al. 2017, 7); we therefore characterize Cupul‐Uicab et al. (2017) effect direction as **mixed (overall null; urban subgroup positive aIRR 1.39)** rather than as the abstract‐only‐era “positive.” Together, Gaylord et al. (2020) and Cupul‐Uicab et al. (2017) expand the OC × functional‐GI evidence from a single Rueda‐Ruzafa‐anchored entry to a three‐paper convergent‐direction signal: Gaylord et al. (2020) newly populate an OC × functional‐GI (celiac) subcell at *n* = 1, and Cupul‐Uicab et al. (2017) raise the OC × functional‐GI overall cell from *n* = 2 to *n* = 3; both contributors carry overall Serious–Moderate ROBINS‐E judgments, so the convergent direction of the OC × functional‐GI signal is best read as preliminary rather than as definitive. Davis et al. (2024) contribute both an OC primary biomarker (*p,p*′‐DDE) and a pyrethroid metabolite (cis‐DBCA from indoor residual spraying) and so appears in both the OC × functional GI and pyrethroid × functional GI cells; we flag this dual‐class assignment in Section 3.1 and again in this paragraph to avoid appearing to multicount. The Japanese cohort (Ueyama et al. 2022) and TwinsUK (Mesnage et al. 2022) contribute SCFA‐functional readouts that overlap into this category.

##### 3.3.3.1 Synthesis

The OC by functional‐GI direction is convergently positive across three contributors (Andalusian IBS, Rueda‐Ruzafa et al. 2023; NYU pediatric celiac, Gaylord et al. 2020; and urban‐stratified Tapachula infant diarrhea, Cupul‐Uicab et al. 2017), but each carries a design ceiling, namely, an ecological proxy, a single‐site pilot with collider risk, and an overall‐null cohort with only an urban‐subgroup signal, respectively, so the convergence is preliminary rather than confirmatory. Two of the four functional‐GI contributors fully support their stated hypothesis, one is partial, and one is overall null (Table 2). The female‐predominant IBS signal (crude OR 2.62) is the most striking and aligns with the sex‐stratified microbiota mechanisms summarized in Section 5.3, but the ecological design caps causal inference.

#### 3.3.4 IBD (Three Distinct Studies, Seven Study‐Contributions, Mostly Positive Direction; *n* Unchanged, the Celiac Reclassification of Gaylord et al. (2020) Keeps It in the Functional‐GI Column Rather Than in the IBD Column Under Our Prespecified PECO Frame)

The two large US cohorts converge on a positive OC‐IBD signal: AHS (Chen, Parks, et al. 2024) ever‐use hazard ratios (HRs) of 1.59 for dieldrin (95% CI, 1.03, 2.44) and 1.61 for toxaphene (95% CI, 1.17, 2.21); Sister Study (Chen, Woo, et al. 2024) DDT‐era residence HR 1.56 (95% CI, 1.06, 2.31). The Faroese CHEF cohort (Hammer et al. 2019, *n* = 5698 with only 37 IBD cases) reports the widest‐CI effect at *p,p*′‐DDT high‐versus‐low risk ratio (RR) 3.04 (95% CI, 1.12, 8.30). Glyphosate × IBD remains empty (*n* = 0). One unifying limitation across all three OC × IBD contributors is the absence of biomarker‐confirmed exposure assessment: AHS and Sister Study rely on recalled self‐report of pesticide use, and the Faroese cohort uses dietary‐vehicle ascertainment of persistent organic pollutant burden rather than serum DDT/DDE measurement at IBD‐incidence interrogation, so the convergent signal across three cohorts is properly read as recall‐based rather than as biomarker‐confirmed evidence.

##### 3.3.4.1 Synthesis

The IBD column is the most internally consistent in the matrix: all three contributors (AHS, Chen, Parks, et al. 2024; Sister Study, Chen, Woo, et al. 2024; Faroese CHEF, Hammer et al. 2019) report positive OC by IBD associations (HR: 1.56–1.61; RR: 3.04), and all three support their stated hypothesis (Table 2). The consistency is genuine but qualified by a shared exposure‐assessment limitation, as none anchored exposure on a contemporaneously measured biomarker; the convergent signal is therefore best framed as three cohorts with shared limitations rather than as three independent confirmations. This is the strongest single‐direction signal in the review and the clearest candidate for biomarker‐anchored replication.

## 4 Gap Matrix Interpretation

**FIGURE 2:**
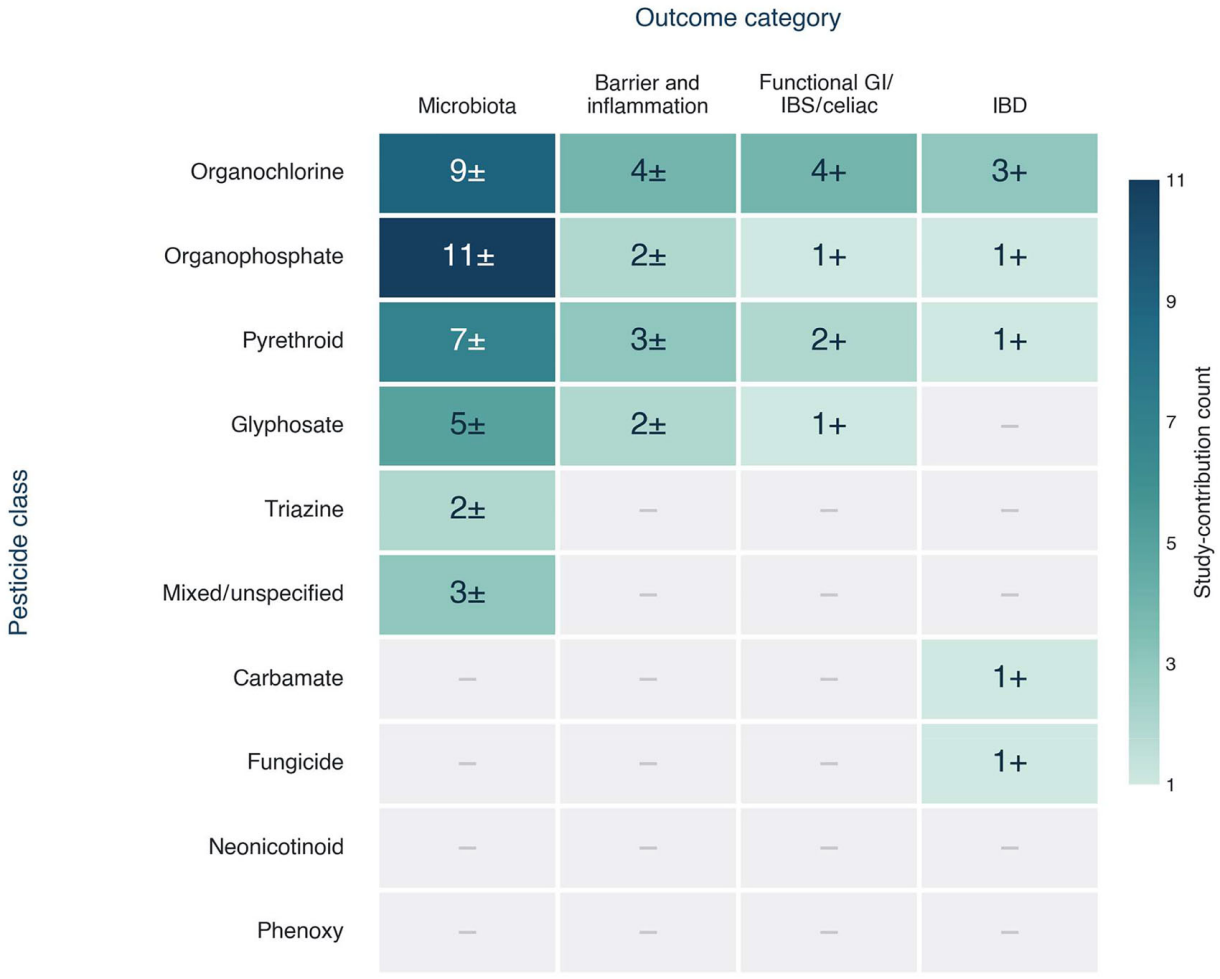
Evidence gap matrix by pesticide class and gastrointestinal outcome. *Note*: Cell shading represents the number of study contributions, and symbols indicate the direction of evidence. Multiclass studies may contribute to more than one pesticide‐class row. The grid displays 19 cells because the four columns collapse six prespecified outcome subcategories and the single organochlorine × functional‐GI cell aggregates two populated subcells; the canonical density of 20 of 60 subcategory cells (33%) is preserved. GI, gastrointestinal; IBD, inflammatory bowel disease; IBS, irritable bowel syndrome; OC, organochlorine.

The gap matrix (Figure 2) renders 20 nonempty cells out of 60 canonical pesticide‐class × outcome combinations (33%), considerably denser than the parallel PFAS gap matrix at ∼10%, reflecting the multiclass nature of pesticide exposure assessment and the contribution of multiclass biomonitoring panels in six newly extracted papers.

The **strongest single‐direction signal** is the OC × IBD cell (*n* = 3 papers, mostly positive direction; CHEF Faroese, AHS, Sister Study). This is the densest convergent cell in the matrix and supports a tentative OC‐pesticide‐class effect on IBD risk in three cohort designs, with the caveat that exposure assessment is recall‐based or biomarker‐decades‐after‐exposure in all three, and we therefore prefer the framing “three cohort designs with shared exposure‐assessment limitations” over “three independent cohort designs.” The **densest single cell** is OP × microbiota (*n* = 11 papers after re‐extraction, mixed direction), but the direction heterogeneity reflects (a) the PEG ambient‐exposure GIS proxy producing 16S taxa‐shift signatures rather than diversity changes (Zhang et al. 2024), (b) the Japanese DAP‐SCFA inverse pattern (Ueyama et al. 2022), (c) the Romanian Tomuța dietary‐modeling case series with no formal regression (Tomuta et al. 2025; Tomuta, Caltea, et al. 2026; Tomuta, Gîtea, et al. 2026), and (d) the newly added MCMC and Hunan hospitalized‐infant cohorts (Dong et al. 2025; Xiang et al. 2025; Yang et al. 2025) whose biomarker‐confirmed multiclass panels report mixed‐direction taxon‐level shifts (e.g., MCMC: pesticide ERS associated with adverse GDM and gestational‐anemia outcomes via *Dorea*‐branch and *Roseburia* microbial mediators; Hunan: phosalone × *Bifidobacterium* coefficient ‐0.620 alongside *o,p*′‐DDT × δ‐HCH × *Pseudomonas* synergistic interaction). These method‐heterogeneity‐driven direction‐mixed cells should be parsed at synthesis stage rather than read as definitive.

### 4.1 Two Confirmed Gaps Requiring Research Priority

#### 4.1.1 Glyphosate × IBD = 0

Despite glyphosate being the most‐used herbicide globally and despite urinary glyphosate detection in 53% of TwinsUK adults (Mesnage et al. 2022) and 100% of US pregnant participants in associated literature, no full‐text‐extracted paper directly examines glyphosate × IBD incidence in human populations. This gap survived the re‐extraction of six newly retrieved papers (none of which assayed glyphosate). It is a clear, prioritizable research target.

#### 4.1.2 Neonicotinoid × Any Outcome = 0

The neonicotinoid class is empty across all four outcome columns (microbiota, barrier and inflammation, functional GI, IBD) in our full‐text extraction. The re‐extraction of the six newly full‐text‐extracted papers (previously abstract‐only) did not populate any neonicotinoid cell: the multiclass MCMC panel (Dong et al. 2025; Yang et al. 2025) measured 29 pesticides but no neonicotinoids; the Hunan 53‐pollutant panel (Xiang et al. 2025) measured 14 herbicides + 9 OCPs + 8 OPs + 7 PAHs + 6 PBDEs + 6 PCBs + 3 pyrethroids without a neonicotinoid subpanel; PIPA (Naspolini et al. 2021), Gaylord et al. (2020), and Cupul‐Uicab et al. (2017) measured OC and pyrethroid metabolites but no neonicotinoids. The empty neonicotinoid row therefore reflects a real evidence gap rather than undercoverage of the abstract‐only papers. Mandatory citation tracing did not identify additional neonicotinoid studies, indicating a substantive evidence absence rather than a search‐strategy artifact.

### 4.2 Sensitivity Analysis: Six Elevated‐Bias Cohorts Excluded From the Primary Direction Summary

Six of the 20 full‐text‐extracted papers received an elevated overall ROBINS‐E judgment: five Serious (Tomuta, Gîtea, et al. 2026; Tomuta et al. 2025; Rueda‐Ruzafa et al. 2023; Hammer et al. 2019; Gaylord et al. 2020) and one **High** (Xiang et al. 2025; D3 selection rated high for the hospitalized‐infant cohort with 92.1% antibiotic exposure), and are excluded jointly in the sensitivity restriction below. For sensitivity‐analysis transparency we identified these six and the cells they populate, and noted that excluding them did not substantially change the direction summary of any cell in this primary gap matrix because each serious‐bias paper is one contributor of three or more in its cell, with one exception flagged below.

**(a) Tomuța (In Vivo;** Tomuta, Gîtea, et al. 2026), ***n* = 26 case series**: contributes to six mixed‐direction study‐contributions (pyrethroid × barrier and inflammation; OP × barrier and inflammation; OC × barrier and inflammation; OC × microbiota; glyphosate × barrier and inflammation; glyphosate × microbiota). Excluding (Tomuta, Gîtea, et al. 2026) reduces those cells’ *n* by 1 each but preserves the mixed summary. **(b) Tomuța (Toxics;** **Tomuta et al**. 2025 **) cohort with uniform background residue exposure and limited microbiome quality control**: contributes to OP × microbiota (one of 11). **(c) Rueda‐Ruzafa (** **Rueda‐Ruzafa et al**. 2023 **) ecological district‐proxy**: contributes to eight cells (four functional GI cells + four microbiota cells) and is the principal evidence for the glyphosate × functional GI cell; excluding (Rueda‐Ruzafa et al. 2023) would empty this cell (*n* → 0). **(d) Hammer Faroese (** **Hammer et al**. 2019 **), *n* = 37 IBD cases**: contributes to OC × IBD (one of three); the cell would remain populated and mostly positive (Sister Study (Chen, Woo, et al. 2024) HR 1.56; AHS (Chen, Parks, et al. 2024) dieldrin HR 1.59) without (Hammer et al. 2019). **(e) Xiang Hunan hospitalized‐infant cohort (** **Xiang et al**. 2025 **), *n* = 304 with 92.1% antibiotic exposure and selection on outcome‐related context**: contributes to OP × microbiota (one of 11), OC × microbiota (one of nine), pyrethroid × microbiota (one of seven), mixed/unspecified × microbiota (one of three), and herbicide × microbiota; excluding (Xiang et al. 2025) reduces those cells’ *n* by 1 each but preserves the mixed summary because of biomarker‐confirmed cohorts in each cell. **(f) Gaylord NYU pediatric celiac case–control (** **Gaylord et al**. 2020 **), *n* = 88 single‐site pilot**: solely populates the OC × functional‐GI (celiac) subcell (one of one); excluding (Gaylord et al. 2020) empties this newly populated celiac subcell entirely (*n* → 0), and the OC × functional‐GI overall cell collapses from *n* = 3 to *n* = 2 (Rueda‐Ruzafa (Rueda‐Ruzafa et al. 2023) and Cupul‐Uicab et al. 2017) with Cupul‐Uicab et al. (2017) positive direction surviving only in the urban subgroup.

The **sensitivity‐restricted matrix** (14 papers excluding the six elevated‐bias cohorts) preserves the OC × IBD primary signal and the OP × microbiota density (which retains the biomarker‐confirmed MCMC, TwinsUK, Japanese, and PEG cohorts), but empties the glyphosate × functional GI cell and the newly populated OC × functional‐GI (celiac) subcell, and reduces the OC × functional GI overall cell from *n* = 3 to a single‐cohort partial signal; these sensitivity‐losses flag the glyphosate × functional‐GI and OC × functional‐GI evidence bases as currently held up by serious‐bias contributors.

A further six papers (Lamichhane et al. 2026; Abimbola et al. 2024; Stanaway et al. 2023; Fagan et al. 2020; Curl et al. 2019; Hyland et al. 2019) remained abstract‐only and were not extracted at full text; they are excluded from the gap matrix and could populate currently empty or low‐density cells if added.

## 5 Discussion

### 5.1 Principal Findings

We mapped 20 full‐text‐extracted human‐population studies onto a 60‐cell pesticide‐class × GI‐outcome matrix; two findings stand out and one absence stands out as loudly. The strongest convergent signal in the matrix is **OC pesticide exposure × IBD**, with three US and Faroese cohorts reporting positive associations under shared exposure‐assessment limitations (all three rely on recall‐based or biomarker‐decades‐after‐exposure ascertainment): the US Sister Study reports an HR of 1.56 (95% CI, 1.06, 2.31) for DDT‐era residential pesticide use (Chen, Woo, et al. 2024); the US AHS reports HRs of 1.59 (95% CI, 1.03, 2.44) for dieldrin and 1.61 (95% CI, 1.17, 2.21) for toxaphene (Chen, Parks, et al. 2024); and the Faroese CHEF cohort reports an RR of 3.04 (95% CI, 1.12, 8.30) for high *p,p*′‐DDT (Hammer et al. 2019). The **densest single cell** is OP × gut microbiota (*n* = 11 papers), but the direction is mixed because effect estimates derive from four incompatible exposure‐assessment regimes (biomarker‐confirmed urinary, biomarker‐confirmed serum, ambient GIS‐proxy, and dietary‐modeled). The most consequential **absences** are the empty cells: no full‐text‐extracted paper directly examines glyphosate × IBD in human populations, and no full‐text‐extracted paper examines neonicotinoids against any of the four GI‐outcome categories, both gaps survived the re‐extraction of six newly retrieved papers. Within the OP × pregnancy‐outcome literature, the MCMC GDM study (Yang et al. 2025) provides the strongest single quantitative finding in our corpus: an ERS adjusted OR of 4.5 (95% CI, 1.4, 13.8; *p* = 0.008) for GDM with an approximately 10‐fold susceptibility in the Prevotella‐low microbiota subgroup and an integrated internal‐validation AUC of 0.83. Because GDM is an adjacent non‐GI endpoint, this finding is best read as the clearest demonstration in our corpus that a measured pesticide mixture can act through a microbiota‐mediated path to a clinical outcome, rather than as direct evidence on any of the four prespecified GI outcome categories; within the GI outcomes proper, the OC × IBD convergence remains the primary signal.

### 5.2 Comparison With Existing Literature

The OC × IBD signal is consistent with the broader epidemiologic literature on persistent organic pollutants and chronic intestinal inflammation, and our extracted evidence base places that literature on a more specific footing in two ways. First, the Faroese cohort (Hammer et al. 2019) reports null associations for the dietary vehicles plausibly carrying OCs in this population (pilot whale meat RR 1.02, 95% CI, 0.48, 2.18; fish RR 1.01; 95% CI, 0.35, 2.91), which weakens the simplest dietary‐vehicle interpretation and strengthens the chemical‐mechanism reading: it is the OC residues themselves, not the food matrix delivering them, that carry the IBD signal in that population. Second, the AHS dose‐tertile analysis (Chen, Parks, et al. 2024) shows elevated HRs across all three intensity‐weighted lifetime‐day tertiles relative to never‐use without a clean monotonic gradient, a pattern compatible with a threshold effect or with the limited within‐cohort variation typical of binary self‐reported ever‐use exposure assessment. For the intensity‐weighted lifetime‐days analysis the elevated HRs span all three tertiles relative to never‐use (for terbufos, tertile 1 HR 1.52 [95% CI, 1.03, 2.24] and tertile 2 HR 1.53 [95% CI, 1.04, 2.25]), a pattern compatible with a threshold rather than a linear dose‐response and convergent with the rodent ADI‐threshold observations in Section 5.3.

The OP × microbiota literature, by contrast, is heterogeneous in a way that reflects exposure‐assessment methodology more than biological signal. The Japanese SCFA cohort (Ueyama et al. 2022) reports a clean inverse association between urinary dialkyl phosphate excretion and fecal acetate (*β* = −24.0, *p* < 0.001; adjusted *R* ^2^ = 0.751 with vegetable‐intake covariate), interpretable as OP burden suppressing SCFA‐producing fermentative function. The TwinsUK shotgun‐metagenomics study (Mesnage et al. 2022) reports glyphosate excretion positively associated with overall species richness, which on its face is the opposite direction from a “pesticide harms microbiome” prior. The MCMC GDM study (Yang et al. 2025) reports a positive direction for the OP‐dominated ERS on adverse pregnancy outcome, mediated by Prevotella‐low and *Dorea*‐branch microbiota subgroups; it is among the strongest reported OP × pregnancy‐outcome quantitative findings in our corpus (adjusted OR 4.5, internal‐validation AUC 0.83). Both effects are real within their respective designs; their apparent contradiction with the TwinsUK richness‐gain pattern dissolves when one notes that the Japanese study measures SCFA chemistry as a functional readout, the MCMC study measures pregnancy clinical outcome with formally mediated microbial subpaths, and the TwinsUK study measures community structure as a compositional readout, and species‐richness gain does not imply functional gain. The PEG ambient‐OP study (Zhang et al. 2024) sits between these: it reports 22 differentially abundant genera and 34 perturbed MetaCyc pathways including reduced B‐vitamin biosynthesis, but uses a 5‐year GIS‐based application‐record proxy at a 500 m radius rather than internal dose. Reading the OP × microbiota cell as a single‐direction summary obscures these structural differences; reading it as four subquestions (biomarker × functional readout, biomarker × compositional readout, biomarker × clinical‐outcome with microbial mediation, ambient proxy × compositional readout) reveals more.

This pattern, in which outcome heterogeneity tracks exposure‐assessment methodology, is the central methodological observation our scoping review can offer. The EFSA Maximum Residue Level (MRL) framework (EC Regulation 396/2005) operates on residues measured in food at the regulatory limit, but the human‐population evidence base operationalizes exposure across at least four distinct levels: (i) urinary metabolite biomarkers reflecting roughly 24‐h internal dose (Mesnage et al. 2022; Ueyama et al. 2022); (ii) blood biomarkers of bioaccumulated lipophilic compounds reflecting years of exposure (Cupul‐Uicab et al. 2017; Davis et al. 2024; Dong et al. 2025; Gaylord et al. 2020; Hammer et al. 2019; Iszatt et al. 2019; Naspolini et al. 2021; Stanaway et al. 2017; Xiang et al. 2025; Yang et al. 2025); (iii) breast‐milk biomonitoring at a critical developmental window (Iszatt et al. 2019); and (iv) recalled exposure decades earlier (Chen, Parks, et al. 2024; Chen, Woo, et al. 2024). None of these four operationalizations maps cleanly onto MRL‐compliant residues at point of consumption, which is what the food‐science side of the chain produces. The Romanian Tomuța cohort (Tomuta et al. 2025) makes this disconnect explicit: in their plant‐rich‐diet population, every food sample tested met EC pesticide‐residue regulatory limits, yet stool 16S analyses showed neuroactive bacterial dysbiosis (reduced *Bifidobacterium* and *Lactobacillus*; elevated *Oscillibacter* and *Alistipes*) and elevated proinflammatory markers. Whether this reflects a true chronic‐low‐dose effect, a confounded plant‐diet pattern, or a methodological artifact of the descriptive design is unresolvable from one cohort, but the observation that “MRL‐compliant” and “microbiota‐undisturbed” are distinct claims is itself the contribution. Beyond EFSA, the parallel US EPA tolerance system, the FAO/WHO Joint Meeting on Pesticide Residues (the international ADI and MRL reference), Japan's Positive List System, and China's national food‐safety risk framework set the regulatory limits against which these exposures should be read (Table 3). Neither *Bifidobacterium* nor *Lactobacillus* has a standardized clinical reference range, so the reductions noted in this cohort are within‐cohort relative shifts rather than deviations from a population norm.

**TABLE 3:** Regulatory and toxicological reference values for focal pesticides.

**Compound (class)**	**US EPA status**	**EU status**	**Year of major restriction**	**ADI or RfD (mg/kg bw/day)**	**Principal human biomarker**	**Note**
DDT (organochlorine)	All food/agricultural uses canceled	Banned; Stockholm Convention POP	US 1972	PTDI 0.01 (JMPR)	Serum *p*,*p*′‐DDE	DDE is the persistent metabolite; still detected in biomonitoring decades after the ban
Dieldrin (OC)	Agricultural uses canceled	Banned (POP)	1987 (US); 2004 (Stockholm POP)	PTDI 0.0001 (aldrin + dieldrin, JMPR)	Serum dieldrin	Legacy bioaccumulated residue
Toxaphene (OC)	Canceled	Banned (POP)	1990 (US); 2004 (Stockholm POP)	No ADI established (banned POP)	Serum toxaphene congeners	Legacy POP
Hexachlorobenzene/HCB (OC)	Not registered as a pesticide	Banned (POP)	2004 (Stockholm POP)	RfD 0.0008 (EPA IRIS)	Serum HCB	Legacy POP; fungicidal use historically
Chlorpyrifos (organophosphate)	Food tolerances revoked 2021, reinstated Dec 2023; EPA 2024 proposed re‐revoking most uses	Approval not renewed (expired)	EU 2020; US 2021	EPA RfD 0.003; EU ADI 0.001	Urinary TCPy; dialkyl phosphates (DAP)	US regulatory status is in flux
Parathion (OP)	All uses canceled (last legal use 2003)	Not approved	2000–2003	RfD 0.006 (EPA, provisional)	Urinary p‐nitrophenol; DAP	Acutely toxic; legacy use
Terbufos (OP)	Registered, Restricted‐Use (RUP)	Not approved	—	Not identified (EPA IRED 2001)	DAP	Soil insecticide
Deltamethrin (pyrethroid)	Registered	Approved	—	ADI 0.01 (JMPR; EU)	Urinary cis‐DBCA; 3‐PBA	cis‐DBCA is a deltamethrin‐specific metabolite
Permethrin (pyrethroid)	Registered (some restricted uses)	Approved (biocidal/limited)	—	EPA RfD 0.25; JMPR ADI 0.05	Urinary 3‐PBA; cis/trans‐DCCA	Also used in vector control / indoor residual spraying
3‐Phenoxybenzoic acid (3‐PBA)	Not a commercial product (metabolite)	Not a commercial product (metabolite)	—	Not applicable (biomarker)	Urinary 3‐PBA	Aggregate pyrethroid‐exposure marker common to deltamethrin, permethrin, and cypermethrin; detected in urine despite limited parent use
Imidacloprid (neonicotinoid)	Registered (under review)	Outdoor agricultural use banned	EU 2018	EPA RfD 0.057; JMPR ADI 0.06	Urinary 5‐hydroxy‐imidacloprid	Neonicotinoid row is empty in the human‐evidence gap matrix; biomarker named for future cohorts
Glyphosate (+ AMPA; glycine herbicide)	Registered (interim decision 2020)	Reauthorized for 10 years (Nov 2023)	EU 2023 renewal	EPA RfD 2; EU ADI 0.5; JMPR ADI 0–1	Urinary glyphosate; AMPA	Most‐used herbicide globally
Atrazine (triazine herbicide)	Registered (under re‐evaluation)	Banned	EU 2004	EPA RfD 0.018	Atrazine mercapturate	EU ban well established; US registration retained
Dicamba (benzoic‐acid herbicide)	Registered; over‐the‐top uses vacated Feb 2024; EPA reregistration proposed	Not approved	—	EPA RfD 0.5; WHO ADI 0.3	Urinary dicamba	Drift‐related restrictions

*Note*: Reference values were summarized from US EPA, EU/EFSA, and FAO/WHO JMPR sources.

Abbreviations: ADI, acceptable daily intake; AMPA, aminomethylphosphonic acid; DAP, dialkyl phosphate; POP, persistent organic pollutant; PTDI, provisional tolerable daily intake; RfD, chronic oral reference dose; TCPy, 3,5,6‐trichloro‐2‐pyridinol.

The intestinal‐barrier and inflammation column is anchored quantitatively by the Norwegian NoMIC cohort (Iszatt et al. 2019), which reports per‐standard‐deviation breast‐milk PBDE‐28 associated with a 24% reduction in infant fecal propionic acid (95% CI, −35, −14) and per‐standard‐deviation PCB‐209 with a 15% reduction in acetic acid (95% CI, −29, −0.4). The same cohort reports per‐standard‐deviation PFOA associated with a 61% increase in propionic acid (95% CI, 35, 87), in the opposite direction. PFOA is a nonpesticide perfluoroalkyl substance and falls outside this review's five‐pesticide‐class focal scope; we retain the PFOA result here as an in‐cohort negative control because the NoMIC analytical panel measured persistent organic pollutants and PFOA in the same milk samples, and the divergent PFOA result reinforces the point that lipophilic organohalogens cannot be lumped together as “pollutants that harm SCFA‐producers.” The South African VHEMBE cohort (Davis et al. 2024) reports per‐10‐fold cis‐DBCA (deltamethrin metabolite) associated with child ear‐infection IRR 1.4 (95% CI, 1.0, 2.1), a mucosal‐immunity readout that lies adjacent to the GI barrier. The functional‐GI / IBS column is dominated by the Andalusian ecological case–control (Rueda‐Ruzafa et al. 2023), with a multivariable‐adjusted total‐population OR for IBS in high‐pesticide‐use districts of 1.26 (printed 95% CI, 1.12, 1.19; mathematical inconsistency in source paper retained verbatim), an unadjusted total‐population OR of 1.16 (95% CI, 1.13, 1.20), and a striking female‐specific crude OR of 2.62 (95% CI, 2.54, 2.70). The female‐specific signal warrants attention because it converges with the sex‐stratified microbiota literature (e.g., gonadal–hormone—microbiota interactions discussed in Mesnage et al. (2022) and Maitre et al. (2018)), but the ecological design ceiling on causal inference is real. The newly added pediatric celiac signal (Gaylord et al. 2020, DDE × celiac aOR 2.04; 95% CI, 1.08, 3.84) and infant‐diarrhea signal (Cupul‐Uicab et al. 2017, urban‐stratified DDE × diarrhea aIRR 1.39; 95% CI, 1.07, 1.80) extend the OC × functional‐GI cell from a single Rueda‐Ruzafa‐anchored entry to a three‐paper convergent direction signal in this column, but both new contributors carry overall Serious–Moderate ROBINS‐E judgments.

The contrast between our findings and the dietary‐intervention literature is informative. The four organic‐diet trials we now have at full text show that dietary substitution reliably reduces urinary pesticide biomarker burden, Bradman et al. (2015) approximately 40% OP metabolite reduction; Curl et al. (2019) urinary 3‐PBA reduction in pregnancy; Hyland et al. (2019) approximately 60%–95% glyphosate and AMPA reduction; Fagan et al. (2020) approximately 70% glyphosate reduction, but only Abimbola et al. (2024) measured a downstream inflammation marker (serum CRP reduction parallel to OP‐metabolite reduction in a Cyprus cluster‐randomized crossover). None of these five trials measured gut microbiota composition, SCFA chemistry, or barrier‐permeability outcomes. This means the existing intervention literature has demonstrated that dietary modification *can* lower pesticide internal dose without ever testing whether that lowering improves microbiome or barrier outcomes, and only one trial (Abimbola et al. 2024) demonstrated a parallel inflammation‐marker reduction; this is an evidence gap that our gap matrix surfaces and that an organic‐diet randomized controlled trial designed with paired microbiota‐and‐barrier outcomes could close.

### 5.3 Mechanism Brief

The 20 human‐population studies that anchor Section 3 are paralleled by an animal and organoid literature that operates at the mechanistic resolution our human evidence cannot reach. We summarize that literature in five short class‐paragraphs, restricted to studies at environmentally relevant doses (approximate ADI/RfD range, not high‐dose toxicology) and emphasizing studies from the past 5 years. Each paragraph cites two representative animal, organoid, or in vitro epithelial‐cell studies and is intended as an interpretive companion to the human‐population evidence rather than as a stand‐alone synthesis.

#### 5.3.1 Organochlorines: Gut Barrier and Immune‐Axis Disruption

Subchronic *p*,*p*′‐DDE exposure in male Sprague‐Dawley rats (2 mg/kg body weight/day by gavage for 21 days) shifts the Firmicutes‐to‐Bacteroidetes ratio and alters lipid‐metabolome–microbiota correlations consistent with a dysbiosis‐driven metabolic phenotype (Liang et al. 2020). Parallel work in adult male C57BL/6 mice exposed to *p,p*′‐DDE (1 mg/kg body weight/day) and β‐hexachlorocyclohexane (β‐HCH; 10 mg/kg body weight/day) for 8 weeks reports OC‐induced expansion of bile‐salt‐hydrolase–active *Lactobacillus* and reduced ileal expression of bile‐acid reabsorption genes, with downstream alterations in hepatic and enteric bile‐acid composition (Liu et al. 2017). These findings provide a coherent mechanistic substrate for the OC × IBD signal observed in the AHS, Sister Study, and Faroese cohorts (Section 5.1): a microbiota–bile acid axis perturbation that is biologically upstream of chronic intestinal inflammation and that operates at exposure ranges within the lipid‐bioaccumulated burden documented in those human cohorts.

#### 5.3.2 Organophosphates: SCFA Suppression and Acetylcholinesterase‐Independent Microbial Effects

Subchronic chlorpyrifos exposure in C57BL/6J mice at the ADI (0.01 mg/kg bodyweight/day) and at 10 × ADI (0.1 mg/kg/day) for 8 weeks disrupts intestinal stem‐cell proliferation and differentiation, lowers fecal butyric acid and indole‐3‐propionic acid, and primes a proinflammatory mucosal immune response, with effects detectable at the regulatory‐relevant ADI dose (Yuan et al. 2025). A Caco‐2/TC7 + SHIME bioreactor coculture exposed to chlorpyrifos reproduces the SCFA‐suppression and tight‐junction‐gene perturbation signal in the absence of acetylcholinesterase substrate, indicating that the gut effect is at least partially independent of the canonical OP neurotoxicity mechanism (Réquilé et al. 2018). This mechanistic profile maps cleanly onto the Japanese DAP × fecal acetate *β* = −24.0 finding in (Ueyama et al. 2022) and onto the perturbed B‐vitamin biosynthesis pathways in (Zhang et al. 2024); it also maps onto the MCMC GDM mediation finding (Yang et al. 2025) where a *Dorea*‐branch microbiota mediates the OP‐dominated ERS‐to‐GDM path. Doubt remains over the dose‐response shape; tertile analyses in the AHS (Chen, Parks, et al. 2024) suggest threshold rather than monotonic dose‐response, which the rodent evidence has only recently begun to probe at ADI‐anchored doses.

#### 5.3.3 Pyrethroids: Barrier and Microbial‐Diversity Effects With Sex‐Dependence

Chronic low‐dose deltamethrin (0.2 mg/kg/day for 8 weeks by oral gavage) in 4‐week‐old female BALB/c mice causes colon‐tissue injury through PRDX1‐inactivation‐driven mitochondrial oxidative stress accompanied by 16S‐rDNA‐detected gut microbial dysbiosis and shortened colon length (Ma et al. 2023). A more recent intergenerational mouse model of preconception parental permethrin exposure (500 µg/kg body weight/day during mating, gestation, and lactation) reports sex‐specific reorganization of microbiota‐tryptophan metabolism in the offspring, with male offspring showing decreased microbial indole and indole‐3‐acetic acid and female offspring showing elevated 5‐hydroxyindole‐3‐acetic acid, alongside microbiota‐transfer‐confirmed causal contributions of *Clostridia* to the metabolic phenotype (Lin et al. 2025). The sex‐stratified mechanistic literature converges with the Andalusian human ecological finding (Rueda‐Ruzafa et al. 2023) of female‐specific elevated IBS prevalence (crude OR 2.62 vs. male OR 1.33) and is biologically consistent with gonadal–hormone—microbiota cross‐talk that the human literature has begun to document.

#### 5.3.4 Glyphosate: Shikimate‐Pathway Disruption in Microbial Commensals

Glyphosate inhibits the shikimate pathway via 5‐enolpyruvylshikimate‐3‐phosphate synthase (EPSPS), which is present in plants and many bacteria but not in mammalian cells. A bioinformatics classification of EPSPS sequences across thousands of species estimates that 54% of species in the core human gut microbiome carry the glyphosate‐sensitive EPSPS class (Class I), with the EPSPS‐insensitive class (Class III, ∼32% of bacterial sequences) enriched in Proteobacteria, providing a specific predicted substrate for differential gut‐commensal susceptibility (Leino et al. 2021). In ovo glyphosate exposure in chick small intestine (10 mg active glyphosate/kg egg mass, embryonic Day 6) reproduces histopathological villus injury and dysregulated tight‐junction gene expression (ZO‐1, ZO‐2, claudin‐1, claudin‐3, JAM2, occludin) alongside elevated proinflammatory cytokines (IFN‐γ, IL‐1β, IL‐6), demonstrating direct intestinal–epithelial sensitivity at concentrations within the residue range encountered in feed (Fathi et al. 2024). This mechanistic substrate is precisely what makes the empty glyphosate × IBD cell in our human gap matrix striking: there is a biologically specific hypothesis (EPSPS‐mediated commensal selection → barrier permeability → IBD) that the population evidence base has not tested. Across these animal and in vitro glyphosate studies, the doses that perturb gut commensals or epithelial integrity (e.g., 10 mg active glyphosate/kg egg mass in ovo (Fathi et al. 2024), and milligram‐per‐kilogram‐per‐day oral regimens in rodent models) lie well above the internal dose attainable from vegetable residues, where glyphosate is detected in roughly one in eight samples at concentrations typically below 0.1 mg/kg (Table S1) against an ADI of 0.5 mg/kg body weight per day (EU). The glyphosate × IBD priority is therefore not merely that no human study exists, but that whether the EPSPS‐mediated mechanism operates at human dietary internal doses remains untested.

#### 5.3.5 Neonicotinoids: Emerging Evidence of Gut‐Microbiome and Barrier Disturbance

Subchronic imidacloprid exposure in male C57BL/6J mice (30 mg/L drinking water for 70 days, equivalent to 10 weeks) impairs gut‐barrier function, increases gram‐negative bacterial overgrowth in colonic contents, and disrupts hepatic/enteric bile‐acid synthesis and reabsorption (Yang et al. 2020). In adult zebrafish, low‐concentration imidacloprid (100 and 1000 µg/L for 21 days) induces intestinal histological injury, elevates intestinal lipopolysaccharide and inflammatory‐cytokine expression, and produces gut microbiota dysbiosis at concentrations within environmental detection ranges (Luo et al. 2021). The mechanistic plausibility extends to the human dietary‐exposure context because vegetable‐residue surveillance data routinely detect neonicotinoid residues. Yet, as Section 3.3 documents, the human‐population evidence base on neonicotinoids × GI outcomes remains empty across all four outcome categories. This is the cleanest example in our review of mechanistic evidence outpacing human‐population evidence; closing the gap requires biomarker‐confirmed cohort designs that include neonicotinoid‐specific urinary metabolites (e.g., 5‐hydroxy‐imidacloprid).

The five‐class brief above is offered as an interpretive companion to the human evidence synthesis, not as a synthesis in its own right. Animal and organoid evidence carry dose‐translation and species‐extrapolation uncertainties that the human‐population evidence does not, and we have deliberately kept the two evidence streams separate in our gap matrix and our direction summaries. Where the two streams converge (OC × barrier permeability, OP × SCFA suppression, pyrethroid × sex‐specific microbiome disturbance) the convergence is informative; where they diverge (glyphosate mechanistic plausibility against the empty human IBD cell, neonicotinoid mechanistic plausibility against an empty human row), the divergence is the research target. Three translational caveats bear emphasis. First, the rodent and organoid doses cited above are best read relative to the ADI and estimated daily intake (EDI): several effects (e.g., chlorpyrifos at the ADI in Yuan et al. 2025) occur at regulatory‐relevant doses, whereas others require supra‐ADI exposure, and Table 3 anchors these comparisons. Second, host genetic susceptibility is an unmeasured modifier in every included human study: IBD risk is shaped by NOD2, IL23R, and intestinal‐barrier‐gene variants, and celiac disease requires the HLA‐DQ2 or HLA‐DQ8 haplotype, so a pesticide effect on barrier or microbiota may be conditional on genotype in ways the population studies could not resolve. Third, interspecies differences in microbiome composition and xenobiotic metabolism limit direct extrapolation, so these convergences are hypotheses for human testing rather than demonstrated human mechanisms.

### 5.4 Strengths and Limitations

#### 5.4.1 Strengths

Three strengths are worth flagging. First, this scoping review maps human‐population pesticide × GI evidence onto a pesticide‐class × outcome‐category gap matrix that includes regulatory‐relevant classes (OP, OC, pyrethroid, glyphosate, neonicotinoid, triazine, mixed/unspecified, carbamate, fungicide) rather than collapsing to “pesticides as a category”; we identified no prior synthesis using this framing. Second, we report gap‐matrix density (20 of 60 cells, 33%) using a transparent operational definition tied to the prespecified PECO frame and the ROBINS‐E appraisal across 20 full‐text‐extracted papers, with multiclass biomonitoring panels resolved at the per‐cell level per the scoping‐review evidence‐density convention. Third, we performed a sensitivity analysis excluding the six elevated‐bias cohorts (Gaylord et al. 2020; Hammer et al. 2019; Rueda‐Ruzafa et al. 2023; Tomuta et al. 2025; Tomuta, Gîtea, et al. 2026; Xiang et al. 2025) and showed that the OC × IBD primary signal and the OP × microbiota density survived their exclusion, while the glyphosate × functional‐GI cell did not, the newly populated OC × functional‐GI (celiac) subcell emptied (*n* = 1 → 0), and the OC × functional‐GI overall cell collapsed from a three‐paper convergent signal to a two‐paper partial signal. This is a transparent statement of which cells of the matrix are robust and which are currently held up by serious‐bias contributors.

#### 5.4.2 Limitations

This scoping review carries several categories of limitation that bear directly on how the gap matrix should be read.

##### 5.4.2.1 Bias in Primary Contributors

Six of the 20 full‐text‐extracted papers received an elevated overall ROBINS‐E judgment (five Serious, one High). The Tomuța (In Vivo) case series (Tomuta, Gîtea, et al. 2026) is descriptive with *n* = 26 and no formal regression; it contributes to six gap‐matrix study‐contributions but does not provide effect estimates. The Tomuța (Toxics) cohort (Tomuta et al. 2025) reports uniformly MRL‐compliant food residues with no within‐cohort exposure variation, which makes any apparent residue–microbiota association unidentifiable from that cohort alone. The Rueda‐Ruzafa Andalusian case–control (Rueda‐Ruzafa et al. 2023) is ecological, with high versus low pesticide‐use district as a binary proxy and minimal covariate adjustment (age, sex, district only); it is the principal evidence for the glyphosate × functional‐GI cell, which empties under sensitivity exclusion. The Hammer Faroese cohort (Hammer et al. 2019) has only 37 IBD cases against a population of 5698, producing the wide CI (RR 3.04; 95% CI, 1.12, 8.30) that drives the OC × IBD signal magnitude. The Xiang Hunan hospitalized‐infant cohort (Xiang et al. 2025) is cross‐sectional with selection on outcome‐related context (hospitalized neonatal‐ward infants with pneumonia, sepsis, or jaundice; 92.1% on antibiotics at sampling); microbiota findings from this cohort should be read as hypothesis‐generating only, given that the gut‐microbiome composition is shaped by clinical disease and antibiotic exposure rather than by pollutant burden. The Gaylord NYU pediatric celiac case–control (Gaylord et al. 2020) is a single‐site convenience pilot in which controls are drawn from the same pediatric GI clinic as cases, creating collider bias on the outcome's screening pathway; it is very wide stratified CIs (e.g., PFOA female OR: 20.6; 95% CI, 1.13, 375; nonpesticide chemical reported here for context only) signal unstable estimates from small subgroup n. We have flagged each in the sensitivity matrix, but readers should weigh these contributors at the appropriate confidence.

##### 5.4.2.2 Abstract‐Only Papers

Six papers (Lamichhane et al. 2026; Abimbola et al. 2024; Stanaway et al. 2023; Fagan et al. 2020; Curl et al. 2019; Hyland et al. 2019) were available only as abstracts and are excluded from the quantitative synthesis and the gap matrix. Their inclusion could populate currently low‐density cells, particularly the OC × infant‐microbiota cell (Lamichhane et al. 2026, prenatal persistent organic pollutants × infant microbiota) and the inflammation‐secondary‐outcome class (Abimbola et al. 2024, a Cyprus organic‐diet trial reporting serum CRP reduction). Such abstract‐based projections are themselves a source of bias and are made cautiously.

##### 5.4.2.3 Methodological Heterogeneity in Microbiome Assays

The 20 full‐text studies differ on three orthogonal axes that block meta‐analytic pooling: 16S rRNA region (V3–V4 in Iszatt et al. (2019) and Yang et al. (2025); V4 in Naspolini et al. (2021), Stanaway et al. (2017), and Zhang et al. (2024); V4–V5 in Xiang et al. (2025); targeted qPCR in Tomuta, Caltea, et al. (2026)); taxonomy database (SILVA, SILVA 138, SILVA 138.1, Greengenes, Greengenes_2013, MetaPhlAn3); and quantification (relative abundance, centered‐log‐ratio transformed, absolute qPCR, shotgun metagenomic species‐level, SCFA targeted chemistry). Direction summaries can be reported under such heterogeneity; quantitative effect‐size pooling cannot. This is a standard scoping‐review constraint per Arksey and O'Malley (2005) and a primary reason we chose the scoping framework rather than a systematic‐review‐with‐meta‐analysis design.

##### 5.4.2.4 Two Papers Excluded at Gap Matrix

Papadopoulou HELIX (Papadopoulou et al. 2019) and Bradman organic‐diet RCT (Bradman et al. 2015) were retrieved at full text but excluded from the gap matrix because their primary outcome is exposure‐biomarker reduction rather than a GI/microbiota/barrier/IBD endpoint. They remain cited in Section 3.2 for exposure‐assessment methodology but do not contribute to outcome‐cell density. Curl et al. (2019), Hyland et al. (2019), and Fagan et al. (2020) sit in a related class (biomarker‐only intervention designs) and would similarly be excluded from outcome‐cell density even at full text; only Abimbola et al. (2024) measured an inflammation‐related secondary outcome, and that single design did not measure microbiota composition.

##### 5.4.2.5 Review‐Process Limitations: Single Database and Single‐Rater Workflow

Only the PubMed search was executed; the protocol‐specified Embase, Web of Science Core Collection, Scopus, and Cochrane CENTRAL searches were not completed, so the evidence base rests on a single primary database offset by mandatory citation tracing and may have missed eligible records indexed only elsewhere. In addition, title/abstract screening, full‐text screening, and data extraction were each performed by one team member, with the lead independently calibrating a 10% screening sample (Cohen's kappa greater than or equal to 0.7) and reappraising a 20% extraction sample, rather than the full dual‐independent screening and extraction recommended by PRISMA‐ScR and JBI guidance, and the kappa calibration covered the title/abstract stage only. This resource‐driven workflow can bias the review toward undercapture of eligible studies and allow individual extraction errors to escape detection; consistent with this, a class‐assignment error found during revision (Gaylord et al. 2020, initially described as a pyrethroid + OC study and corrected to OC [DDE] only) prompted a recheck of all pesticide‐class assignments, and the gap‐matrix density (20 of 60 cells) should be read as sensitive to such single‐rater class assignments.

##### 5.4.2.6 Scoping Framework Constraints

Per the scoping review framework (Arksey and O'Malley 2005) and PRISMA‐ScR (Tricco et al. 2018), we did not perform formal effect‐size pooling, did not assign Grading of Recommendations Assessment, Development and Evaluation (GRADE) levels of evidence to individual cells, and did not produce overall certainty ratings. Our direction‐summary categories (mostly positive, mostly inverse, mixed, no data) are descriptive of cell‐level evidence under the exposure‐class × outcome‐category mapping we operationalized, not synthetic causal claims.

##### 5.4.2.7 Toxicological Framing, Threshold of Toxicological Concern, and Natural Pesticides

Four further limitations bear on regulatory interpretation. First, the included studies rarely translate exposure into an EDI comparable against an ADI or RfD; we surface available exposure values in Tables 2 and 3 but cannot reconstruct intake‐versus‐ADI margins for most cohorts. Second, no included study applied a threshold of toxicological concern (TTC) or Cramer‐class screening lens, the appropriate regulatory‐toxicology frame for chronic low‐level dietary residues, so its absence is a genuine gap rather than a stylistic choice. Third, regulatory status is heterogeneous across the source jurisdictions and shifts over time (Table 3). Fourth, as developed in Section 5.6, natural and biopesticide residues and plant‐innate defensive metabolites were measured by no included study, so this synthesis characterizes synthetic‐pesticide evidence specifically.

### 5.5 Public Health and Policy Implications

The two primary cell‐level absences in the gap matrix translate directly into two prioritizable research targets, and the methodological pattern we documented translates into one design recommendation for the next generation of dietary‐intervention trials.

#### 5.5.1 Glyphosate × IBD: Priority for Prospective Cohort Design

Despite glyphosate being the most‐used herbicide globally, despite urinary glyphosate detection in 53% of TwinsUK adults (Mesnage et al. 2022), and despite the recent EU renewal of glyphosate approval through December 15, 2033, no full‐text‐extracted paper in this scoping review directly examines glyphosate × IBD incidence in a human population. The Sister Study (Chen, Woo, et al. 2024) and AHS (Chen, Parks, et al. 2024) are well‐placed to add glyphosate‐specific analyses given their existing IBD ascertainment, within the existing recall‐based exposure framework; biomarker‐anchored analyses would require new urinary glyphosate/AMPA collection, for example, via a Sister Study or AHS substudy, or via the planned EFSA pesticide biomarker harmonization effort. Either path would produce evidence directly relevant to future regulatory review of glyphosate.

#### 5.5.2 Neonicotinoids: A Priority for Human‐Population Studies

The neonicotinoid row of the gap matrix is empty across all four outcome columns and across the six papers that remain abstract‐only (Lamichhane et al. 2026; Abimbola et al. 2024; Stanaway et al. 2023; Fagan et al. 2020; Curl et al. 2019; Hyland et al. 2019); the re‐extraction of six newly retrieved papers (Dong et al. 2025; Xiang et al. 2025; Yang et al. 2025; Naspolini et al. 2021; Gaylord et al. 2020; Cupul‐Uicab et al. 2017) did not populate any neonicotinoid cell because none of the assayed multipollutant panels (29‐pesticide MCMC; 53‐pollutant Hunan; 49‐chemical PIPA) included a neonicotinoid subpanel, and the celiac and diarrhea cohorts (Gaylord et al. 2020; Cupul‐Uicab et al. 2017) measured OC chemicals only. This is striking because animal evidence for neonicotinoid × gut microbiome perturbation has accumulated over the past 5 years (Section 5.3) and because neonicotinoid residues are routinely detected in vegetable surveillance data. The empty row therefore reflects a real evidence gap rather than undercoverage of the abstract‐only papers; the evidence base on neonicotinoids × GI outcomes starts from zero.

#### 5.5.3 Organic‐Diet RCT Design Recommendation: Pair Biomarker Reduction With Microbiota Outcomes

The five organic‐diet biomarker trials we identified (Bradman et al. 2015; Curl et al. 2019; Hyland et al. 2019; Fagan et al. 2020; Abimbola et al. 2024) demonstrate that organic‐diet substitution reduces urinary OP, glyphosate, and pyrethroid biomarkers across 6‐day to 24‐week windows by approximately 40%–95%. Only Abimbola et al. (2024) measured an inflammation‐related secondary outcome (parallel reduction in serum CRP); none measured gut microbiota, SCFA chemistry, or barrier‐permeability markers as a primary outcome. The Bradman et al. (2015) design (crossover with washout) is methodologically transferable: a future RCT in the same class, sized for paired stool 16S or shotgun metagenomics with SCFA chemistry as primary outcomes, would test the causal link between residue‐burden reduction and microbiome recovery that the current literature can only assume. The Stanaway et al. (2023) oral‐microbiome paper, although outside Section 3.2 organic‐diet trial set (it is an observational oral‐microbiome outcome study in pesticide‐exposed farmworker children, not a biomarker‐reduction intervention), demonstrates that microbiota outcomes can be feasibly added in pesticide‐exposed pediatric populations and would complement intervention designs. Stratifying such a trial by baseline pesticide burden (high vs. low detection panel) would address the threshold‐effect question that the AHS tertile analyses (Chen, Parks, et al. 2024) raised.

#### 5.5.4 Food‐Analysis Side: Couple Residue‐Removal Efficiency With Intake‐Weighted Exposure Modeling

The food‐science literature on washing and cooking removal efficiency is well‐developed (representative processing factors by treatment and pesticide class are compiled in Table S2) but is rarely coupled with consumption modeling on the epidemiologic side. A practical implication is that exposure‐assessment methodology in future cohorts should record vegetable‐preparation behavior alongside FFQ intake, particularly for OP and pyrethroid residues where surface‐rinse removal efficiency is high. This recommendation is methodological rather than mechanistic, but it bears directly on how MRL‐compliant residues at point of sale translate to internal dose at the urinary biomarker level; this is the gap that motivated this scoping review at the outset.

### 5.6 Synthetic Versus Natural and Biopesticide Residues

A structural limitation of the present evidence base, and of the residue‐monitoring system that feeds it, is that both were built around synthetic active ingredients, whereas natural pesticides warrant equal attention. Three categories of nonsynthetic exposure are largely absent from the human‐population literature we mapped. First, botanical and microbial biopesticides approved for organic and conventional production (pyrethrins, azadirachtin, rotenone, spinosad, copper and sulfur fungicides, and *Bacillus thuringiensis*) are applied to vegetables but are not quantified by most routine multiresidue methods; organic produce is therefore not residue‐free, and several natural actives carry their own toxicological profile (rotenone is a mitochondrial complex‐I inhibitor, and copper accumulates in soil and tissue). Second, plant‐innate defensive metabolites intrinsic to many vegetables, notably glucosinolates and their isothiocyanate breakdown products in cruciferous vegetables and glycoalkaloids such as solanine in solanaceous crops, are pharmacologically active on the gut epithelium and microbiota and are modulated by the same washing, peeling, and cooking steps that affect synthetic residues, yet they are rarely comeasured in dietary‐pesticide epidemiology. Third, none of the 20 included human studies measured natural‐pesticide exposure, so the present gap matrix is, strictly, a matrix of synthetic‐pesticide evidence. We flag this asymmetry rather than resolve it: a complete account of vegetable‐borne chemical exposure and gut health will require exposure‐assessment frameworks that quantify natural actives and plant secondary metabolites alongside synthetic residues, a priority gap also listed in Section 5.4 and the Conclusion section.

## 6 Integrated Synthesis and Broader Context

Two findings organize this scoping review and a third absence organizes what comes next. The most consistent positive signal in the evidence base is the convergence of three cohorts on an OC × IBD association (Sister Study HR 1.56 for DDT‐era residential exposure; AHS dieldrin HR 1.59 and toxaphene HR 1.61; Faroese CHEF *p,p*′‐DDT RR 3.04), all three carrying a shared exposure‐assessment limitation in that none anchored exposure on a contemporaneously measured biomarker (Chen, Parks, et al. 2024; Chen, Woo, et al. 2024; Hammer et al. 2019). The most quantitatively striking newly added contribution is the MCMC pregnancy cohort (Yang et al. 2025), in which a multipesticide ERS adjusted OR for GDM reached 4.5 (95% CI, 1.4, 13.8) with an integrated internal‐validation AUC of 0.83 and a formal microbiota‐mediated path through a Prevotella‐low subgroup and a *Dorea*‐branch mediator. Against these signals stand two clear evidence gaps: no full‐text‐extracted paper directly examines glyphosate × IBD in human populations, and no full‐text‐extracted paper measures neonicotinoid biomarkers against any of the four GI‐outcome categories. The strongest signals and the cleanest gaps emerged from the same matrix; they should be read together rather than in isolation.

The 20‐of‐60 cell density (33%) is meaningfully higher than the 28% reported at the preliminary stage, and that increase is informative methodologically. Six newly retrieved full‐text papers contributed to the increment: the MCMC companion cohorts in Nanjing (Dong et al. 2025; Yang et al. 2025), the Hunan hospitalized‐infant 53‐pollutant panel (Xiang et al. 2025), the Brazilian PIPA pilot (Naspolini et al. 2021), the NYU pediatric celiac case–control (Gaylord et al. 2020), and the Tapachula boys‐only DDT‐and‐diarrhea cohort (Cupul‐Uicab et al. 2017). Three of these (Dong et al. 2025; Xiang et al. 2025; Yang et al. 2025) are multiclass biomarker panels measuring 29–53 chemicals across OC, OP, pyrethroid, herbicide, triazine, chloroacetamide, and conazole rows; the matrix gain is therefore not driven by new outcome categories but by exposure breadth that single‐class designs cannot attain. The increment also exposes a methodological dichotomy that the earlier corpus blurred. The cohorts now anchoring our matrix divide cleanly into biomarker‐confirmed designs (TwinsUK, NoMIC, Japanese SCFA, MCMC, PIPA, Tapachula, NYU; *n* = 7 designs) and self‐reported or recall‐based exposure designs (AHS, Sister Study; the two largest cohorts in the corpus by sample size). Effect‐direction summaries pool poorly across this dichotomy because the two design classes operationalize different exposure constructs at different time scales, even when they label the chemical class identically.

The risk‐of‐bias distribution across the 22 ROBINS‐E‐rated units (0 Low, 5 Low‐to‐Moderate, 11 Moderate, 5 Serious, 1 High, 0 Critical) is centered on Moderate, which is the realistic and expected pattern for environmental epidemiology when full‐cohort prospective designs with biomarker‐confirmed exposure are scarce. The 22 ROBINS‐E appraisals comprise the 20 full‐text‐extracted synthesis studies plus two cite‐only methodology references that fall outside the locked PICO (the HELIX exposure‐determinant study, Papadopoulou et al. 2019, and the Bradman et al. 2015 organic‐diet biomarker‐reduction trial), both rated Low‐to‐Moderate and appraised for transparency though neither populates a gap‐matrix cell. The six elevated‐bias cohorts (five Serious, one High) carry distinct mechanisms: ecological proxy (Rueda‐Ruzafa et al. 2023), small case series with no formal regression (Tomuta, Gîtea, et al. 2026), uniformly MRL‐compliant residue exposure with no within‐cohort variation (Tomuta et al. 2025), wide‐CI rare‐outcome cohort (Hammer et al. 2019), single‐site GI‐clinic pilot with collider‐bias controls (Gaylord et al. 2020), and hospitalized‐infant cohort with 92.1% antibiotic exposure (Xiang et al. 2025). Sensitivity exclusion of all six did not collapse the OC × IBD primary signal but did empty the glyphosate × functional‐GI cell and reduced the OC × functional‐GI cell to a single‐cohort partial signal. This is a transparent statement of which cells are robust and which are currently held up by serious‐bias contributors, and it does more interpretive work than a simple direction summary would.

The Mechanism Brief section sketches the epidemiology‐to‐biology bridge as follows: the OC × IBD epidemiologic signal sits over a microbiota–bile acid axis demonstrated in C57BL/6 mice (Liu et al. 2017); the OP × microbiota epidemiologic signal sits over a stem‐cell and SCFA‐suppression mechanism demonstrated at the ADI dose in mice (Yuan et al. 2025); the pyrethroid × functional‐GI female‐specific human signal sits over an intergenerational tryptophan‐axis mechanism with sex‐specific offspring phenotypes (Lin et al. 2025); and the empty glyphosate × IBD and neonicotinoid × any‐GI cells sit over biologically specific mechanistic predictions (EPSPS‐mediated commensal selection (Leino et al. 2021); imidacloprid‐driven gram‐negative overgrowth and bile‐acid disruption (Yang et al. 2020)) that the human evidence base has not tested. Where mechanism and epidemiology converge the convergence is informative; where mechanism outpaces epidemiology, the divergence is the research target.

This scoping picture sits coherently against the broader environmental‐epidemiology and gut‐microbiota literature on persistent organic pollutants and trace‐metal exposures. The PFAS‐and‐gut‐microbiota literature has converged on an SCFA‐suppression and bile‐acid disruption signal in early life (a pattern visible in the NoMIC PFOA in‐cohort comparison (Iszatt et al. 2019)) that runs parallel to but distinct from the OC pesticide signal we summarize here. The heavy‐metals‐and‐infant‐microbiome literature reflected in PIPA (Naspolini et al. 2021) suggests that pesticide effects can be of smaller magnitude than comeasured metal effects in cohorts where both are quantified, which has direct implications for how multipollutant panel data should be reported in single‐class reviews. The air‐pollution‐and‐gut‐microbiome literature is at an earlier mapping stage but follows the same biomarker‐versus‐proxy dichotomy our matrix surfaces. The contribution our matrix makes against this adjacent body of work is the operational translation of “there is a literature” into “here are the populated cells, the empty cells, and the bias‐weighted populated cells,” which the adjacent fields have not yet rendered at this granularity.

This review carries six categories of limitation that bear on how the matrix should be read. First, we synthesized under the scoping framework rather than as a systematic‐review‐with‐meta‐analysis, and we did not assign GRADE certainty levels to individual cells. Second, we restricted to English peer‐reviewed literature, which underrepresents Chinese, Latin American, and African epidemiologic evidence on dietary pesticide exposure. Third, the two MCMC companion papers (Dong et al. 2025; Yang et al. 2025) are sample‐overlapping by design and are reported as one cohort with two outcomes for sample‐size accounting; future meta‐analytic work should propagate this overlap explicitly. Fourth, a separate six papers remain abstract‐only (PDFs retrieved but not yet full‐text extracted) and are excluded from the gap matrix; cell density may rise further at the next iteration, particularly in the OC × infant‐microbiota and inflammation‐secondary‐outcome cells. Fifth, exposure‐assessment heterogeneity across urinary biomarker, blood biomarker, breast‐milk biomarker, ambient proxy, and recalled use precludes effect‐size pooling and is itself one of the central methodological observations of the review. Sixth, only PubMed was searched (the protocol‐specified Embase, Web of Science, Scopus, and Cochrane CENTRAL searches were not run) and screening and extraction were single‐rater with partial calibration, so the evidence base may undercapture eligible studies and individual extraction errors.

## 7 Conclusion

This scoping review mapped 28 papers (20 full‐text‐extracted human studies) onto a 60‐cell pesticide‐class × GI‐outcome matrix and rendered 20 cells (33%) populated, with the strongest convergent signal at OC × IBD across three cohorts and the strongest single quantitative finding at the MCMC pesticide ERS for GDM (adjusted OR: 4.5; 95% CI, 1.4, 13.8; internal‐validation AUC 0.83 with quantitative microbiota‐mediated path estimate). The GDM result is an adjacent non‐GI endpoint, reported here as a microbiota‐mediation exemplar rather than as a within‐scope GI outcome. Against these positive signals, the matrix surfaces three priority gaps for primary research. The first is glyphosate × IBD, which remains empty across the corpus despite glyphosate being the most‐used herbicide globally and despite the recent EU renewal of glyphosate approval through December 15, 2033; the Sister Study and AHS are well‐positioned to add glyphosate‐specific analyses within their existing IBD ascertainment, and a biomarker‐anchored substudy would produce evidence directly relevant to that regulatory file. The second is neonicotinoid × any GI outcome, which remains empty across all four outcome columns and across the six newly retrieved papers, and which requires biomarker‐confirmed cohort designs that include neonicotinoid‐specific urinary metabolites such as 5‐hydroxy‐imidacloprid. The third is biomarker‐anchored prospective pediatric cohorts that pair contemporaneously measured pesticide exposure with functional GI outcomes; the existing biomarker‐confirmed pediatric evidence is held up by single‐site pilots and one boys‐only cohort. The translational framing matters because vegetable consumption guidance and pesticide‐residue exposure framing currently sit in noncommunicating literatures, and closing the cells identified here would let dietary recommendations be anchored on residue‐and‐microbiome evidence rather than on residue‐only exposure inference. Throughout, the cell‐level direction summaries are descriptive and should not be read as cause‐and‐effect claims; and because natural and biopesticide residues and plant‐innate metabolites were measured by none of the included studies, the priorities above should be pursued with exposure‐assessment frameworks that quantify natural actives alongside synthetic residues and that situate measured intakes against ADI and threshold‐of‐toxicological‐concern benchmarks.

## Author Contributions

**Chengqian Ye**: conceptualization, methodology, data curation, writing – original draft. **Qingjie Wu**: writing – original draft, methodology, formal analysis, visualization. **Zhaochu Wang**: writing – review and editing, data curation, validation. **Cai Zhang**: writing – review and editing, conceptualization, methodology, supervision, project administration, visualization.

## Funding

The authors received no specific funding for this work.

## Ethics Statement

Not applicable, as this scoping review only synthesized published literature.

## Conflicts of Interest

The authors declare no conflicts of interest.

## Supporting information




**Supporting Information**: crf370622‐sup‐0001‐SuppMat.docx

## Data Availability

The evidence table, risk‐of‐bias assessments (22 cohorts), gap‐matrix cell data, full‐text retrieval status, search log, and decision log are available from the corresponding author upon reasonable request.
