# Supplementary material for: Pesticide Residues, Gut Microbiota, and Gastrointestinal Outcomes: A Scoping Review of Human Evidence
Source: Compr Rev Food Sci Food Saf. 2026 Aug 23;25(5):e70622. doi: 10.1111/1541-4337.70622 (PMC13500746; doi:10.1111/1541-4337.70622)
Supplement: Supplementary file 1 [file CRF3-25-e70622-s001.docx]

**Supplemental Material**

Pesticide residues, gut microbiota, and gastrointestinal outcomes: a scoping review of human evidence

**Contents**

[Table S1. Pesticide residue occurrence in vegetables from official monitoring programs, by pesticide class and vegetable group.	2](#_Toc237881567)

[Table S2. Household and processing reduction of pesticide residues on vegetables (% reduction or processing factor, PF), by treatment and pesticide class.	4](#_Toc237881568)

[Table S3. Full electronic search strategies, by database.	6](#_Toc237881569)

[Table S4. PRISMA-ScR (2018) checklist.	10](#_Toc237881570)

[Figure S1. Conceptual pathway linking vegetable pesticide residues to gastrointestinal outcomes, and the two non-communicating literatures the review bridges.	14](#_Toc237881571)

# Table S1. Pesticide residue occurrence in vegetables from official monitoring programs, by pesticide class and vegetable group.

| **Pesticide class** | **Leafy greens**  **(spinach, lettuce, kale)** | **Cruciferous**  **(cabbage, broccoli)** | **Root/bulb**  **(potato, carrot, onion)** | **Solanaceous/fruiting**  **(tomato, eggplant, pepper)** | **Legumes/other**  **(beans, peas)** |
| --- | --- | --- | --- | --- | --- |
| **Organophosphates (OPs)** | DF: 41.7% (chlorpyrifos, spinach) [S5]  Median: NR  Max: 3.00 mg/kg (chlorpyrifos) [S5]  MRL exc.: NR | DF: 33.3% (chlorpyrifos, cabbage) [S5]  Median: NR  Max: 2.40 mg/kg (chlorpyrifos) [S5]  MRL exc.: NR | DF: 25% (chlorpyrifos, onion) [S5]  Median: NR  Max: 2.12 mg/kg (chlorpyrifos) [S5]  MRL exc.: NR | DF: NR  Median: NR  Max: NR  Chlorpyrifos among top violative/ARfD drivers [S1, S3] | DF: NR  Median: NR  Max: NR  MRL exc.: NR |
| **Pyrethroids** | DF: 33.3% (cypermethrin, spinach) [S5]  Median: NR  Max: 0.040 mg/kg (cypermethrin) [S5]  MRL exc.: NR | DF: 25% (cypermethrin, cabbage) [S5]  Median: NR  Max: 0.030 mg/kg (cypermethrin) [S5]  MRL exc.: NR | DF: 16.7% (cypermethrin, onion) [S5]  Median: NR  Max: 0.014 mg/kg (cypermethrin) [S5]  MRL exc.: NR | DF: NR  Median: NR  Max: 0.23 mg/kg (bifenthrin, eggplant) [S7]  MRL exc.: NR | DF: NR  Median: NR  Max: NR  MRL exc.: NR |
| **Neonicotinoids** | DF: 45.6% (imidacloprid, lettuce); 38.7% (spinach) [S4]  Median: NR  Max: 1.6 ppm (acetamiprid, spinach) [S4]  MRL exc.: NR | DF: NR  Median: NR  Max: NR  Acetamiprid most-detected substance, EU programme [S1] | DF: NR  Median: NR  Max: NR  MRL exc.: NR | DF: 39/139 acetamiprid; 11/139 imidacloprid (tomato) [S7]  Range: 0.017-0.347 mg/kg (acetamiprid) [S7]  Max: 0.38 mg/kg (imidacloprid) [S7]  MRL exc.: NR | DF: NR  Median: NR  Max: NR  MRL exc.: NR |
| **Organochlorines (OC; DDT/DDE/HCB)** | DF: 75% (p,p'-DDD, spinach) [S5]  Median: NR  Max: 0.64 mg/kg (p,p'-DDD) [S5]  MRL exc.: NR | DF: 83.3% (p,p'-DDD, cabbage) [S5]  Median: NR  Max: 0.60 mg/kg (a-endosulfan) [S5]  MRL exc.: NR | DF: 2.7% DDT/DDD/DDE (potato) [S2]; 50% p,p'-DDD (onion) [S5]  Median: NR  Max: 0.010 mg/kg (p,p'-DDD, onion) [S5]  MRL exc.: NR | DF: NR  Median: NR  Max: NR  MRL exc.: NR | DF: NR  Median: NR  Max: NR  MRL exc.: NR |
| **Glyphosate*** | DF: ~12% (fresh fruit/veg category) [S6]  Median: NR  Max: 0.085 mg/kg (fresh veg) [S6]  MRL exc.: NR (veg); 0.3% overall, mainly buckwheat [S1] | DF: ~12% (fresh fruit/veg category) [S6]  Median: NR  Max: 0.085 mg/kg [S6]  MRL exc.: NR | DF: ~12% (fresh fruit/veg category) [S6]  Median: NR  Max: 0.085 mg/kg [S6]  MRL exc.: NR | DF: ~12% (fresh fruit/veg category) [S6]  Median: NR  Max: 0.085 mg/kg [S6]  MRL exc.: NR | DF: beans 40%, lentils 33%, peas 22% [S6]  Median: NR  Max: 8.6 mg/kg (beans) [S6]  MRL exc.: 0.7% (pulses; 5/770) [S6] |
| **All classes - program-level anchors** | EFSA EU-coordinated programme (2022): 1.6% of samples exceeded MRL; 98.4% within legal limits [S1]. USDA PDP (2022): 0.53% exceeded EPA tolerance; 27.6% no detectable residue [S2]. US FDA (FY2022) vegetables: violative residues in 6.5% domestic / 12.6% imported samples; no residue in 38.6% domestic / 42.7% imported [S3]. | | | | |

**Notes:** DF, detection frequency (% of samples with residue ≥ LOQ); Median, typical/median residue level; Max, maximum residue level reported; MRL, maximum residue level; ARfD, acute reference dose; LOQ, limit of quantification; NR, not reported (no sourced value available); ppm, parts per million (= mg/kg).

Official monitoring programs (EFSA, US FDA, USDA PDP) report results by commodity or by individual pesticide and do not publish a pesticide-class × vegetable-group occurrence matrix; class × group cells are therefore drawn from program reports where available [S1–S3] and otherwise from peer-reviewed monitoring surveys [S4–S7], with each value individually cited. No values were interpolated or estimated; cells without a sourced value are marked NR.

Survey-derived values (S4–S7) originate from specific national markets and analytical scopes (US PDP/TDS, Tanzania, Saudi Arabia, Canada) and are not interchangeable with EU/US program-wide rates; they indicate magnitude, not program statistics. MRL/tolerance exceedance is a regulatory-compliance metric, distinct from detection frequency.

*Glyphosate is poorly captured by standard multiresidue (QuEChERS-based) screens because of its high polarity, and requires dedicated single-residue / EURL-QuPPe LC-MS/MS methods [S1, S6]; its occurrence in vegetables is therefore under-ascertained in many programs, and most program glyphosate exceedances arise in grains (e.g., buckwheat) and pulses rather than vegetables [S1, S6]. FAO/WHO JMPR sets ADI/ARfD and recommends Codex MRLs but does not conduct occurrence monitoring, so no occurrence value here is attributed to JMPR.

**Sources:** [S1] EFSA. EFSA J 2024;22(4):e8753 (doi:10.2903/j.efsa.2024.8753). [S2] USDA-AMS. Pesticide Data Program Annual Summary, CY2022 (2024). [S3] US FDA. Pesticide Residue Monitoring Program, FY2022 Report (2024). [S4] Craddock et al. Environ Health 2019;18:7 (doi:10.1186/s12940-018-0441-7). [S5] Mahugija et al. Int J Anal Chem 2017;2017:4676724 (doi:10.1155/2017/4676724). [S6] Kolakowski et al. J Agric Food Chem 2020;68:5201-5211 (doi:10.1021/acs.jafc.9b07819). [S7] Selim et al. Molecules 2023;28:1343 (doi:10.3390/molecules28031343).

# Table S2. Household and processing reduction of pesticide residues on vegetables (% reduction or processing factor, PF), by treatment and pesticide class.

| **Treatment** | **Organophosphates (e.g., chlorpyrifos, dimethoate, malathion)** | **Pyrethroids (e.g., cypermethrin, deltamethrin)** | **Neonicotinoids (e.g., imidacloprid, acetamiprid)** | **Organochlorines (DDT/DDE, HCB, lindane)** | **Glyphosate** |
| --- | --- | --- | --- | --- | --- |
| **Washing (running/tap water)** | 20–77% reduction (brinjal, cauliflower, okra) [Kumari 2008]; methamidophos ≈65% after 1 h, plateau thereafter [Tan & Li 1998] | 10–70% reduction (least effective single step) [Chauhan 2014] | Imidacloprid 3.5% (tap water) [Shayanrad 2025]; 12–43% on leafy veg [Kwon 2009] | Surface-localized OC partly removed; up to 100% of DDT in cabbage with washing alone [Terfe 2023] | NR (systemic; poorly washed off — PFs generally near 1) [Yigit 2019] |
| **Washing (2% NaCl / salt)** | Within 20–89% range for chemical-bath washes [Satpathy 2012] | Saline + detergent washes 34–60% (more effective than tap water) [Chauhan 2014] | Imidacloprid 8.8% (NaCl) [Shayanrad 2025] | Higher reduction than tap water for surface OC [Islam 2022] | NR |
| **Washing (NaHCO₃ / baking soda)** | Within 20–89% chemical-bath range [Satpathy 2012] | NaHCO₃ baths among more effective chemical washes (>tap water) [Chung 2018] | Imidacloprid 50.4% (NaHCO₃, most effective wash) [Shayanrad 2025] | 0.1% NaHCO₃ effective for surface OC; part of best combined wash [Islam 2022] | NR |
| **Washing (vinegar / acetic acid)** | Within 20–89% chemical-bath range [Satpathy 2012] | Acidic baths effective for surface SP residues [Chung 2018] | Imidacloprid 30.0% (acetic acid) [Shayanrad 2025] | Acetic/vinegar effective for surface OC; 2% vinegar second-best single wash [Islam 2022] | NR |
| **Peeling** | Highly effective for surface (non-systemic) residues; “most effective” for topmost-layer residues [Yigit 2019] | 60–100% reduction (most effective single treatment) [Chauhan 2014] | Limited: systemic, distributes into flesh — peeling removes only surface fraction [Xiao 2025] | Effective: lipophilic OC concentrate in waxy peel/skin [Yigit 2019] | Limited (systemic, translocated) [Xiao 2025] |
| **Blanching** | Reduces residues more effectively than washing for many OPs [Chung 2018] | Contributes to 75–98% range achieved by cooking/thermal steps [Chauhan 2014] | Leafy-veg blanching lower reduction for some actives vs washing [Yang 2022] | Partial; thermal/volatilization-dependent [Yigit 2019] | NR |
| **Boiling / cooking** | 52–100% (OP, market & spiked veg) [Satpathy 2012]; 32–100% (brinjal/cauliflower/okra) [Kumari 2008] | Cooking eliminates 75–98% of residues [Chauhan 2014] | Imidacloprid up to 94% after boiling (chard) [Kwon 2009]; running water 77% / boiling 59.5% leafy veg [Yang 2022] | Washing+boiling → 100% DDT reduction in cabbage [Terfe 2023] | Thermal degradation of hydrophilic/low-log Kₒₖ actives [Xiao 2025] |
| **Frying / cooking in oil** | Variable; reductions lower when cooked in oil (≈45%) [Chauhan 2014] | Reductions lower in oil (≈45%) than aqueous cooking [Chauhan 2014] | NR | May concentrate (lipophilic OC partition into oil/fat; PF can exceed 1) [Xiao 2025] | NR |
| **Surface vs systemic residue behaviour** | Mostly surface/contact (chlorpyrifos) but some systemic (dimethoate) — washing less effective for systemic [Xiao 2025] | Lipophilic, surface/cuticle-bound → washing/peeling effective [Chauhan 2014] | Systemic; translocated into tissue — surface washing only partially effective [Lu et al. 2017; Xiao 2025] | Lipophilic, surface- and wax-associated; persist, concentrate in fat [Yigit 2019] | Highly systemic, water-soluble; not removed by surface washing [Yigit 2019] |

*Notes: Values are approximate percentage reductions in residue concentration (% reduction) relative to untreated produce, or, where indicated, processing factors (PF). PF = residue concentration in processed commodity ÷ residue concentration in raw commodity; PF < 1 indicates a decrease and PF > 1 (e.g., during drying or oil cooking of lipophilic compounds) indicates concentration. % reduction ≈ (1 − PF) × 100.*

*Reported values are matrix-, active-substance-, and method-dependent (commodity, residue age and location [surface vs systemic], pesticide physicochemical properties [log Kₒₖ, water solubility, volatility], wash time/temperature, and analytical method), so ranges across studies are wide and not directly additive across sequential steps. NR = no sourced value identified for that cell (absence of a value does not imply zero effect).*

*Abbreviations: OP, organophosphate; SP, synthetic pyrethroid; OC, organochlorine; DDT, dichlorodiphenyltrichloroethane; DDE, dichlorodiphenyldichloroethylene; HCB, hexachlorobenzene; NaCl, sodium chloride; NaHCO₃, sodium bicarbonate; Kₒₖ, octanol–water partition coefficient; PF, processing factor; NR, not reported.*

*For curated, FoodEx2-coded processing factors across pesticide/commodity combinations, see the EU database of processing factors for pesticide residues (Zincke 2022, EFSA Supporting Publication EN-7453; subsequent updates Kittelmann 2024 EN-8738, 2025 EN-9477).*

# Table S3. Full electronic search strategies, by database.

The strategy combines three Boolean concept blocks with AND, as described in Methods §2.3: Block 1, pesticide-class and chemical names plus controlled-vocabulary headings; Block 2, vegetable and dietary-source terms plus headings; Block 3, gut-microbiota, intestinal-barrier/inflammation, functional-GI, and inflammatory-bowel-disease terms plus headings. A publication-year limit of 2010 to April 2026 and an English-language limit were applied at the database level. Syntax is adapted per database (PubMed [MeSH]/[tiab]; Embase Emtree/.mp; Web of Science Core Collection TS=; Scopus TITLE-ABS-KEY; Cochrane CENTRAL). Strings are faithful expansions of the concept blocks in §2.3 and are provided for reproducibility.

| **Database** | **Complete Boolean search string (three concept blocks combined with AND)** | **Hits / date of search** |
| --- | --- | --- |
| **PubMed** | #1 Block 1 - Pesticide class & chemicals  ("Pesticides"[Mesh] OR "Organophosphates"[Mesh] OR "Pyrethrins"[Mesh]  OR "Neonicotinoids"[Mesh] OR "Hydrocarbons, Chlorinated"[Mesh]  OR "Glyphosate"[Mesh] OR pesticid*[tiab] OR organophosphate*[tiab]  OR organophosphorus[tiab] OR chlorpyrifos[tiab] OR malathion[tiab]  OR "dialkyl phosphate*"[tiab] OR pyrethroid*[tiab] OR permethrin[tiab]  OR deltamethrin[tiab] OR "3-phenoxybenzoic acid"[tiab]  OR neonicotinoid*[tiab] OR imidacloprid[tiab] OR clothianidin[tiab]  OR thiamethoxam[tiab] OR organochlorine*[tiab] OR DDT[tiab] OR DDE[tiab]  OR hexachlorocyclohexane[tiab] OR dieldrin[tiab] OR toxaphene[tiab]  OR glyphosate[tiab] OR AMPA[tiab])  AND  #2 Block 2 - Vegetable / dietary source  ("Vegetables"[Mesh] OR "Diet"[Mesh] OR "Diet Surveys"[Mesh]  OR "Food, Organic"[Mesh] OR vegetable*[tiab] OR "leafy green*"[tiab]  OR cruciferous[tiab] OR produce[tiab] OR "dietary intake"[tiab]  OR "food frequency"[tiab] OR FFQ[tiab] OR "duplicate diet"[tiab]  OR "organic diet"[tiab] OR "organic food"[tiab] OR "dietary exposure"[tiab])  AND  #3 Block 3 - Gut microbiota / barrier / functional-GI / IBD  ("Gastrointestinal Microbiome"[Mesh] OR "Dysbiosis"[Mesh]  OR "Inflammatory Bowel Diseases"[Mesh] OR "Irritable Bowel Syndrome"[Mesh]  OR "Celiac Disease"[Mesh] OR "Intestinal Mucosa"[Mesh]  OR "Gastrointestinal Diseases"[Mesh] OR "gut microbiot*"[tiab]  OR microbiome[tiab] OR microbiota[tiab] OR 16S[tiab] OR metagenomic*[tiab]  OR "short-chain fatty acid*"[tiab] OR "intestinal permeability"[tiab]  OR zonulin[tiab] OR "lipopolysaccharide-binding protein"[tiab]  OR calprotectin[tiab] OR "gut barrier"[tiab] OR IBS[tiab]  OR "irritable bowel"[tiab] OR "functional dyspepsia"[tiab]  OR celiac[tiab] OR coeliac[tiab] OR IBD[tiab]  OR "inflammatory bowel"[tiab] OR Crohn*[tiab] OR colitis[tiab])  AND  #4 Limits  (("2010/01/01"[Date - Publication] : "2026/04/30"[Date - Publication])  AND English[Language])  Search: #1 AND #2 AND #3 AND #4 | 207 (primary query, pre-check 2026-04-27); 263 unique PMIDs after deduplication across 1 primary + 12 supplementary queries (§2.3; Fig. 1) |
| **Embase (Ovid/Elsevier)** | #1 Block 1 - Pesticide class & chemicals  ('pesticide'/exp OR 'organophosphate pesticide'/exp OR 'pyrethroid'/exp  OR 'neonicotinoid'/exp OR 'organochlorine pesticide'/exp OR 'glyphosate'/exp  OR pesticid*:ti,ab OR organophosphate*:ti,ab OR organophosphorus:ti,ab  OR chlorpyrifos:ti,ab OR malathion:ti,ab OR 'dialkyl phosphate*':ti,ab  OR pyrethroid*:ti,ab OR permethrin:ti,ab OR deltamethrin:ti,ab  OR '3 phenoxybenzoic acid':ti,ab OR neonicotinoid*:ti,ab  OR imidacloprid:ti,ab OR clothianidin:ti,ab OR thiamethoxam:ti,ab  OR organochlorine*:ti,ab OR DDT:ti,ab OR DDE:ti,ab  OR hexachlorocyclohexane:ti,ab OR dieldrin:ti,ab OR toxaphene:ti,ab  OR glyphosate:ti,ab OR AMPA:ti,ab)  AND  #2 Block 2 - Vegetable / dietary source  ('vegetable'/exp OR 'diet'/exp OR 'organic food'/exp  OR vegetable*:ti,ab OR 'leafy green*':ti,ab OR cruciferous:ti,ab  OR produce:ti,ab OR 'dietary intake':ti,ab OR 'food frequency':ti,ab  OR FFQ:ti,ab OR 'duplicate diet':ti,ab OR 'organic diet':ti,ab  OR 'organic food':ti,ab OR 'dietary exposure':ti,ab)  AND  #3 Block 3 - Gut microbiota / barrier / functional-GI / IBD  ('intestine flora'/exp OR 'dysbiosis'/exp OR 'inflammatory bowel disease'/exp  OR 'irritable colon'/exp OR 'celiac disease'/exp  OR 'intestine mucosa'/exp OR 'gut microbiot*':ti,ab OR microbiome:ti,ab  OR microbiota:ti,ab OR 16S:ti,ab OR metagenomic*:ti,ab  OR 'short chain fatty acid*':ti,ab OR 'intestinal permeability':ti,ab  OR zonulin:ti,ab OR 'lipopolysaccharide binding protein':ti,ab  OR calprotectin:ti,ab OR 'gut barrier':ti,ab OR IBS:ti,ab  OR 'irritable bowel':ti,ab OR 'functional dyspepsia':ti,ab  OR celiac:ti,ab OR coeliac:ti,ab OR IBD:ti,ab  OR 'inflammatory bowel':ti,ab OR Crohn*:ti,ab OR colitis:ti,ab)  AND  #4 Limits  ([2010-2026]/py AND [english]/lim AND [embase]/lim)  Search: #1 AND #2 AND #3 AND #4  (.mp = title, abstract, keyword fields; /exp = Emtree exploded) | Protocol-specified string; search not executed (review limited to PubMed; see Methods §2.3 and Limitations §5.4). |
| **Web of Science Core Collection** | #1 Block 1 - Pesticide class & chemicals  TS=(pesticid* OR organophosphate* OR organophosphorus OR chlorpyrifos  OR malathion OR "dialkyl phosphate*" OR pyrethroid* OR permethrin  OR deltamethrin OR "3-phenoxybenzoic acid" OR neonicotinoid*  OR imidacloprid OR clothianidin OR thiamethoxam OR organochlorine*  OR DDT OR DDE OR hexachlorocyclohexane OR dieldrin OR toxaphene  OR glyphosate OR AMPA)  AND  #2 Block 2 - Vegetable / dietary source  TS=(vegetable* OR "leafy green*" OR cruciferous OR produce  OR "dietary intake" OR "food frequency" OR FFQ OR "duplicate diet"  OR "organic diet" OR "organic food" OR "dietary exposure")  AND  #3 Block 3 - Gut microbiota / barrier / functional-GI / IBD  TS=("gut microbiot*" OR microbiome OR microbiota OR 16S OR metagenomic*  OR "short-chain fatty acid*" OR "intestinal permeability" OR zonulin  OR "lipopolysaccharide-binding protein" OR calprotectin OR "gut barrier"  OR IBS OR "irritable bowel" OR "functional dyspepsia" OR celiac  OR coeliac OR IBD OR "inflammatory bowel" OR Crohn* OR colitis)  Combine: #1 AND #2 AND #3  Refine: Publication Years = 2010-2026; Languages = English  (Web of Science Core Collection; TS = Topic field = title, abstract,  author keywords, Keywords Plus) | Protocol-specified string; search not executed (review limited to PubMed; see Methods §2.3 and Limitations §5.4). |
| **Scopus** | #1 Block 1 - Pesticide class & chemicals  TITLE-ABS-KEY(pesticid* OR organophosphate* OR organophosphorus  OR chlorpyrifos OR malathion OR "dialkyl phosphate*" OR pyrethroid*  OR permethrin OR deltamethrin OR "3-phenoxybenzoic acid"  OR neonicotinoid* OR imidacloprid OR clothianidin OR thiamethoxam  OR organochlorine* OR DDT OR DDE OR hexachlorocyclohexane  OR dieldrin OR toxaphene OR glyphosate OR AMPA)  AND  #2 Block 2 - Vegetable / dietary source  TITLE-ABS-KEY(vegetable* OR "leafy green*" OR cruciferous OR produce  OR "dietary intake" OR "food frequency" OR FFQ OR "duplicate diet"  OR "organic diet" OR "organic food" OR "dietary exposure")  AND  #3 Block 3 - Gut microbiota / barrier / functional-GI / IBD  TITLE-ABS-KEY("gut microbiot*" OR microbiome OR microbiota OR 16S  OR metagenomic* OR "short-chain fatty acid*" OR "intestinal permeability"  OR zonulin OR "lipopolysaccharide-binding protein" OR calprotectin  OR "gut barrier" OR IBS OR "irritable bowel" OR "functional dyspepsia"  OR celiac OR coeliac OR IBD OR "inflammatory bowel" OR Crohn* OR colitis)  AND  #4 Limits  PUBYEAR > 2009 AND PUBYEAR < 2027  AND LIMIT-TO(LANGUAGE,"English")  Full: #1 AND #2 AND #3 AND #4 | Protocol-specified string; search not executed (review limited to PubMed; see Methods §2.3 and Limitations §5.4). |
| **Cochrane CENTRAL** | #1 Block 1 - Pesticide class & chemicals  (pesticid* OR organophosphate* OR chlorpyrifos OR malathion  OR "dialkyl phosphate*" OR pyrethroid* OR permethrin OR deltamethrin  OR "3-phenoxybenzoic acid" OR neonicotinoid* OR imidacloprid  OR organochlorine* OR DDT OR DDE OR dieldrin OR toxaphene  OR glyphosate OR AMPA):ti,ab,kw  AND  #2 Block 2 - Vegetable / dietary source (emphasis: organic-diet trials)  (vegetable* OR "leafy green*" OR cruciferous OR produce  OR "organic diet" OR "organic food" OR "organic produce"  OR "dietary intervention" OR "dietary exposure" OR FFQ):ti,ab,kw  AND  #3 Block 3 - Gut microbiota / barrier / functional-GI / IBD  ("gut microbiot*" OR microbiome OR microbiota OR "short-chain fatty acid*"  OR "intestinal permeability" OR zonulin OR calprotectin OR "gut barrier"  OR IBS OR "irritable bowel" OR celiac OR coeliac OR IBD  OR "inflammatory bowel" OR Crohn* OR colitis):ti,ab,kw  AND  #4 Limits  Custom date range 2010 to 2026; Trials (CENTRAL)  Search: #1 AND #2 AND #3 AND #4  (Cochrane CENTRAL used as a backstop for organic-diet intervention trials;  :ti,ab,kw = title/abstract/keyword fields) | Protocol-specified string; search not executed (organic-diet backstop; review limited to PubMed; see §2.3 and §5.4). |

Notes. MeSH = Medical Subject Headings; tiab = title/abstract field (PubMed); Emtree = Embase subject headings; /exp = exploded heading; .mp / :ti,ab(,kw) = multi-field (title, abstract, keyword) search; TS = Topic field (Web of Science); FFQ = food-frequency questionnaire; AMPA = aminomethylphosphonic acid; IBS = irritable bowel syndrome; IBD = inflammatory bowel disease. Block 1 covers the five focal pesticide classes (organophosphates, pyrethroids, neonicotinoids, organochlorines, glyphosate) with class names, representative active ingredients, and validated urinary/serum biomarkers; carbamate, phenoxy, and triazine terms were not added as focal exposures and were captured only when co-measured (§2.2). PubMed was the only database searched; the strings for Embase, Web of Science, Scopus, and Cochrane CENTRAL were specified in the protocol but not executed (§2.3, §5.4), so no yields are reported for them. Database-specific syntactic adaptations are recorded in the search log. Grey-literature sources (EFSA, U.S. EPA Office of Pesticide Programs, FAO/WHO JMPR, China NCFSRA) and mandatory forward/backward citation tracing (Web of Science, Connected Papers, Research Rabbit) supplemented the database searches (§2.3).

# Table S4. PRISMA-ScR (2018) checklist.

Checklist reproduced from Tricco AC, Lillie E, Zarin W, et al. PRISMA Extension for Scoping Reviews (PRISMA-ScR): Checklist and Explanation. Ann Intern Med. 2018;169(7):467-473. doi:10.7326/M18-0850. The PRISMA-ScR comprises 20 essential reporting items and 2 optional items, numbered 1 to 22 below; all are mapped to their location in the manuscript. “NA” denotes items not applicable to a scoping review under this framework.

| **Item #** | **Section** | **Checklist item** | **Description** | **Reported** | **Location in manuscript (§ / page)** |
| --- | --- | --- | --- | --- | --- |
| 1 | **TITLE** | **Title** | Identify the report as a scoping review. | **Yes** | Title (“… a scoping review of human evidence”) |
| 2 | **ABSTRACT** | **Structured summary** | Provide a structured summary that includes (as applicable) background, objectives, eligibility criteria, sources of evidence, charting methods, results, and conclusions that relate to the review questions and objectives. | **Yes** | Abstract (Background / Methods / Results / Conclusion) |
| 3 | **INTRODUCTION** | **Rationale** | Describe the rationale for the review in the context of what is already known. Explain why the review questions/objectives lend themselves to a scoping review approach. | **Yes** | §1 Introduction (paras 1-4) |
| 4 |  | **Objectives** | Provide an explicit statement of the questions and objectives being addressed with reference to their key elements (e.g., population, concepts, and context) or other relevant key elements used to conceptualize the review questions and/or objectives. | **Yes** | §1 Introduction (final para; PECO chain and three objectives) |
| 5 | **METHODS** | **Protocol and registration** | Indicate whether a review protocol exists; state if and where it can be accessed (e.g., a Web address); and if available, provide registration information, including the registration number. | **Partial** | §2.1 (protocol finalised 2026-04-27; archived in the project repository: predefined protocol, search strategy, and extraction template). Not registered in PROSPERO; protocol available on request, no registration number. |
| 6 |  | **Eligibility criteria** | Specify characteristics of the sources of evidence used as eligibility criteria (e.g., years considered, language, and publication status), and provide a rationale. | **Yes** | §2.2 Eligibility criteria (PECO i-vi; 2010-April 2026; English peer-reviewed only; exclusions a-e) |
| 7 |  | **Information sources** | Describe all information sources in the search (e.g., databases with dates of coverage and contact with authors to identify additional sources), as well as the date the most recent search was executed. | Partial | §2.3 (PubMed executed; protocol-specified Embase, Web of Science Core Collection, Scopus, and Cochrane CENTRAL not executed; grey literature; citation tracing). Table S3; Limitations §5.4. |
| 8 |  | **Search** | Present the full electronic search strategy for at least 1 database, including any limits used, such that it could be repeated. | **Yes** | §2.3 (concept blocks); executed PubMed strategy plus protocol-specified (unexecuted) strings for the other four databases in Supplementary Table S3; search log |
| 9 |  | **Selection of sources of evidence** | State the process for selecting sources of evidence (i.e., screening and eligibility) included in the scoping review. | **Yes** | §2.4 (EndNote/Zotero dedup; Rayyan single-rater screening with a 10% double-screened calibration check, Cohen's kappa ≥0.7; full-text assessment). Fig. 1 PRISMA-ScR flow. |
| 10 |  | **Data charting process** | Describe the methods of charting data from the included sources of evidence (e.g., calibrated forms or forms that have been tested by the team before their use, and whether data charting was done independently or in duplicate) and any processes for obtaining and confirming data from investigators. | **Yes** | §2.4 (predefined extraction template; one row per exposure × outcome in the evidence table; controlled pesticide-class vocabulary; lead re-checked a sample) |
| 11 |  | **Data items** | List and define all variables for which data were sought and any assumptions and simplifications made. | **Yes** | §2.4 and Table 2 columns (cohort, country, design/population, pesticide class, exposure-assessment method, outcome category, key result, ROBINS-E rating, finding vs. hypothesis) |
| 12 |  | **Critical appraisal of individual sources of evidence** | If done, provide a rationale for conducting a critical appraisal of included sources of evidence; describe the methods used and how this information was used in any data synthesis (if appropriate). | **Yes** | §2.4 (ROBINS-E for observational studies; Cochrane RoB 2.0 for organic-diet trials; microbiome quality-control grading at three levels). Used in sensitivity analysis (§4). |
| 13 |  | **Synthesis of results** | Describe the methods of handling and summarizing the data that were charted. | **Yes** | §2.5 Synthesis (no pooling; narrative mapping; pesticide-class × outcome gap matrix; Mechanism Brief; methodological-challenges synthesis) |
| 14 | **RESULTS** | **Selection of sources of evidence** | Give numbers of sources of evidence screened, assessed for eligibility, and included in the review, with reasons for exclusions at each stage, ideally using a flow diagram. | **Yes** | §3 (28 included: 20 full-text-extracted, 2 cite-only, 6 abstract-only) and Figure 1 PRISMA-ScR flow diagram (263 unique PMIDs; rescue/exclude lists) |
| 15 |  | **Characteristics of sources of evidence** | For each source of evidence, present characteristics for which data were charted and provide the citations. | **Yes** | §3, §3.1-3.2, and Table 2 (study-level characteristics for all 20 full-text-extracted studies) |
| 16 |  | **Critical appraisal within sources of evidence** | If done, present data on critical appraisal of included sources of evidence (see item 12). | **Yes** | §4 and §6 (ROBINS-E across 22 appraisals = 20 synthesis studies + 2 out-of-PICO cite-only references; distribution 0 Low, 5 Low-to-Moderate, 11 Moderate, 5 Serious, 1 High, 0 Critical); Table 2 per-study ROBINS-E rating |
| 17 |  | **Results of individual sources of evidence** | For each included source of evidence, present the relevant data that were charted that relate to the review questions and objectives. | **Yes** | §3.3 (outcome evidence by category with per-study effect estimates and 95% CIs) and Table 2 |
| 18 |  | **Synthesis of results** | Summarize and/or present the charting results as they relate to the review questions and objectives. | **Yes** | §3.1-3.3, §4 Gap-matrix interpretation, and Figure 2 (20/60 cells populated; OC × IBD signal; glyphosate × IBD and neonicotinoid rows empty) |
| 19 | **DISCUSSION** | **Summary of evidence** | Summarize the main results (including an overview of concepts, themes, and types of evidence available), link to the review questions and objectives, and consider the relevance to key groups. | **Yes** | §5.1 Principal findings, §5.2 Comparison with literature, §6 Integrated synthesis, §5.5 Public-health/policy implications |
| 20 |  | **Limitations** | Discuss the limitations of the scoping review process. | **Yes** | §5.4 Limitations and §6 Integrated Synthesis |
| 21 |  | **Conclusions** | Provide a general interpretation of the results with respect to the review questions and objectives, as well as potential implications and/or next steps. | **Yes** | §7 Conclusion (three priority research gaps; translational framing) |
| 22 | **FUNDING** | **Funding** | Describe sources of funding for the included sources of evidence, as well as sources of funding for the scoping review. Describe the role of the funders of the scoping review. | **Partial** | End matter: Funding (no specific grant received) and Role of the funder (no role). Funding of individual included studies not separately tabulated. |

Notes. § = manuscript section. Reported = Yes (fully addressed) / Partial (addressed but incomplete relative to the item) / No / NA (not applicable to a scoping review). Nineteen items are reported Yes. Three are Partial: item 5 (Protocol and registration), because the protocol exists and is archived in the project repository but the review was not registered in PROSPERO and carries no registration number; item 7 (Information sources), because PubMed was the only database executed; and item 22 (Funding), because funding of the individual included studies is not separately tabulated. No item is rated No or NA.

# Figure S1. Conceptual pathway linking vegetable pesticide residues to gastrointestinal outcomes, and the two non-communicating literatures the review bridges.

*A) The review bridges food-science evidence on pesticide residues, MRL compliance, and processing with gut-health evidence on microbiota, bias appraisal, and GI outcomes.*

*(B) A hypothesis-generating pathway is proposed in which dietary residues may influence gut microbiota, intestinal barrier function, inflammation, and downstream functional GI disorders or IBD; this figure does not imply causal proof.*

*(C) Exposure assessment methods capture different time windows, ranging from urinary metabolites to serum OC, breast milk, and long-term self-report.*

*(D) The human evidence map summarizes signals, limited evidence, and gaps across pesticide classes and GI outcomes. Overall, 20 of 60 evidence cells are populated, with priority gaps for glyphosate–IBD and neonicotinoids–GI outcomes. No new data are presented.*

*Abbreviations: GI, gastrointestinal; IBD, inflammatory bowel disease; MRL, maximum residue limit; OC, organochlorine; RoB, risk of bias.*
